# Methods of Modeling of Strongly Correlated Electron Systems

**DOI:** 10.3390/nano13020238

**Published:** 2023-01-05

**Authors:** Roman Kuzian

**Affiliations:** 1 Donostia International Physics Center (DIPC), Paseo Manuel de Lardizabal 4, 20018 Donostia, Basque Country, Spain; roman.kuzian@dipc.org; 2 Frantsevich Institute for Problems of Materials Science NASU, Krzhizhanovskogo 3, 03180 Kiev, Ukraine

**Keywords:** strongly correlated solids, charge-transfer insulators, Löwdin downfolding, canonical transform, Schrieffer–Wolff transform, Hubbard model, Anderson model, cuprates

## Abstract

The discovery of high-$T_{c}$ superconductivity in cuprates in 1986 moved strongly correlated systems from exotic worlds interesting only for pure theorists to the focus of solid-state research. In recent decades, the majority of hot topics in condensed matter physics (high-$T_{c}$ superconductivity, colossal magnetoresistance, multiferroicity, ferromagnetism in diluted magnetic semiconductors, etc.) have been related to strongly correlated transition metal compounds. The highly successful electronic structure calculations based on density functional theory lose their predictive power when applied to such compounds. It is necessary to go beyond the mean field approximation and use the many-body theory. The methods and models that were developed for the description of strongly correlated systems are reviewed together with the examples of response function calculations that are needed for the interpretation of experimental information (inelastic neutron scattering, optical conductivity, resonant inelastic X-ray scattering, electron energy loss spectroscopy, angle-resolved photoemission, electron spin resonance, and magnetic and magnetoelectric properties). The peculiarities of (quasi-) 0-, 1-, 2-, and 3- dimensional systems are discussed.

## 1. Introduction

The strongly correlated transition metal compounds remain a focus of attention in the condensed matter scientific community since they may show high-$T_{c}$ superconductivity in quasi-two-dimensional cuprates [1,2], frustrated magnetism in low-dimensional cuprates and other materials [3,4,5,6], colossal magnetoresistance and observation of Griffiths phase in manganites [7,8,9,10], multiferroism  [11,12], or spin liquid behavior [13,14,15], as well as many other interesting properties. The interpretation of the vast experimental information depends on the calculation of various response functions. The task becomes highly non-trivial for the transition metal compounds because of the strong correlations in the *d*- or *f*-electron shell of the transition metal ions. The difficulty is due to the necessity to take into account the many-body effects. These effects cannot be described by finite orders of the perturbation theory because of high (macroscopically large) degeneracy of the unperturbed state. Thus, the summation of terms up to the infinite order of perturbation theory or non-perturbative theoretical methods should be used [16].

The commonly used approach has serious limitations: for an interpretation of an experiment, the response function is calculated for a model, the parameters of the model being taken from a fit to experimental data. The model thus describes the dynamics of the studied system in an energy range that is relevant for the calculated response. However, the interrelation between responses on different energy scales is missing in this approach. In particular, the models describing the optical response (the energy range of several eV) has usually nothing in common with the spin-Hamiltonians that describe the magnetic properties of the same compound (the energy range of several meV).

The aim of this review is to outline a systematic theoretical approach for the calculation of physical properties and response functions for different kinds of transition metal compounds on the basis of modern understanding of electronic structure and of the role of correlations in electron motion. We introduce the models and methods that are exploited for the description of strongly correlated systems. Simple analytically solvable finite system Hamiltonians demonstrate the basis physics of strongly correlated systems.

## 2. Electron Correlations. Finite Systems

### 2.1. Hydrogen Molecule

As we mentioned in the Introduction, the description of transition metal compounds demands the account of correlation in the motion of *d* and *f*-electrons [16]. The simplest system that demonstrates the role of correlation effects is the electronic structure of the hydrogen molecule. We adopt the Born–Oppenheimer approximation, where the nuclei positions are fixed in the points $R_{1}$, $R_{2}$. So, we consider the motion of two electrons that experience an attraction by protons and a repulsion from each other.

In the mean field Hückel theory [17,18,19,20], it is assumed that every electron moves in a self-consistent external field $V_{SCF}\left(r\right)$ that is a sum of the attraction to the nuclei and the repulsion from the second electron averaged over the positions of this electron. On the basis of orthogonalized functions of the hydrogen atom ground state $\phi_{i}\left(r\right)=\phi \left(r-R_{i}\right)$, the mean-field theory Hamiltonian has the form (in the second quantization)

(1) $${\hat{H}}_{SCF}=\varepsilon_{SCF}\sum_{i,s}a_{i,s}^{†}a_{i,s}+\hat{t},\;\hat{t}=-t\sum_{s}\left(a_{1,s}^{†}a_{2,s}+a_{2,s}^{†}a_{1,s}\right)$$

where the diagonal and non-diagonal matrix elements are (t>0)

(2) $$\varepsilon_{SCF}=\langle \phi_{i}\left(r\right)|-\frac{1}{2}▵+V_{SCF}\left(r\right)|\phi_{i}\left(r\right)\rangle ,\;-t=\langle \phi_{1}\left(r\right)|-\frac{1}{2}▵+V_{SCF}\left(r\right)|\phi_{2}\left(r\right)\rangle$$

operator $a_{i,s}^{†}$ creates an electron with orbital function $\phi_{i}\left(r\right)$ and spin index s=↑,↓. Without loss of generality, we may assume the orthogonality of basis functions $\langle \phi_{i}\left(r\right)|\phi_{j}\left(r\right)\rangle =\delta_{ij}$; then creation and annihilation operators satisfy usual fermionic commutation relations $a_{i,s}a_{j,s}^{†}+a_{j,s}^{†}a_{i,s}=\delta_{ij}$ (for the generalization for the non-orthgonal basis case see chapter 2 of the P. Fulde book [16]).

**Figure 1:**
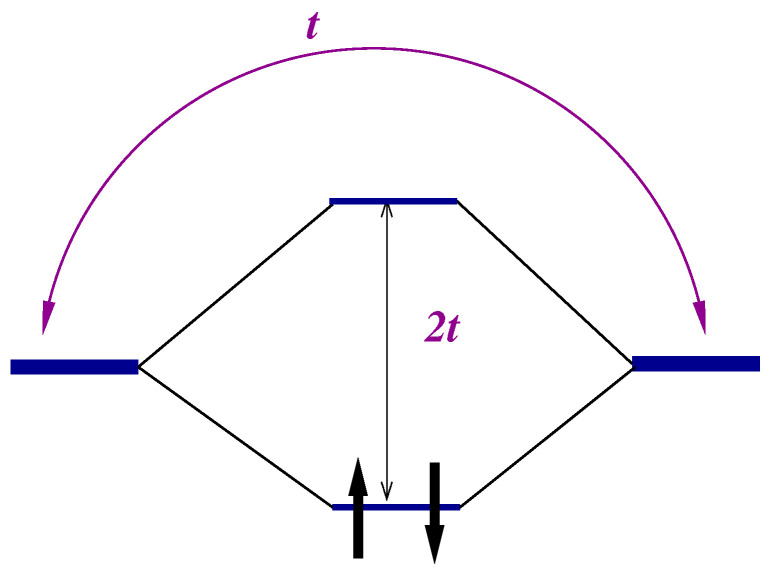
Mean field description of hydrogen molecule electronic structure.

Within the approximations made, the Hamiltonian ${\hat{H}}_{SCF}$ (1) has (for each value of the spin projection *s*) two eigenfunctions (bonding and antibonding orbitals, see Figure 1) with energies $e_{\pm }=\varepsilon_{SCF}\pm t$

(3) $$|\psi_{\pm }\rangle =\frac{1}{\sqrt{2}}\left[\phi_{1}\left(r\right)\mp \phi_{2}\left(r\right)\right].$$

In the ground state, both electrons occupy the lowest energy bonding orbital $|\psi_{-}\rangle$. The electrons should have opposite spins. So, two electron ground state function has the form

(4) $$\begin{array}{cc} |g_{SCF}\rangle & =a_{-,↑}^{†}a_{-,↓}^{†}|vac\rangle =\frac{1}{\sqrt{2}}\left(|s_{1}\rangle +|s_{2}\rangle \right)\\ \; & =\frac{1}{\sqrt{8}}\left[\phi_{1}\left(r_{1}\right)\phi_{2}\left(r_{2}\right)+\phi_{2}\left(r_{1}\right)\phi_{1}\left(r_{2}\right)+\phi_{1}\left(r_{1}\right)\phi_{1}\left(r_{2}\right)+\phi_{2}\left(r_{1}\right)\phi_{2}\left(r_{2}\right)\right]\left(\alpha_{1}\beta_{2}-\beta_{1}\alpha_{2}\right), \end{array}$$

(5) $$\begin{array}{cc} |s_{1}\rangle & =\frac{1}{\sqrt{2}}\left[a_{1,↑}^{†}a_{2,↓}^{†}+a_{2,↑}^{†}a_{1,↓}^{†}\right]|vac\rangle ,\;|s_{2}\rangle =\frac{1}{\sqrt{2}}\left[a_{1,↑}^{†}a_{1,↓}^{†}+a_{2,↑}^{†}a_{2,↓}^{†}\right]|vac\rangle . \end{array}$$

The spinors α,β refer to spin up and spin down, respectively. We see that electrons move independently in the mean field approximation. The probability of finding them on the same site (it is given by the square of the coefficient of the so-called ionic configurations that are given by the terms of the form $a_{i,↑}^{†}a_{i,↓}^{†}|vac\rangle$) equals the probability to finding the electrons on different sites (square of the coefficient of the terms $a_{1,↑}^{†}a_{2,↓}^{†}|vac\rangle$). This means the absence of *the correlations* in the electron motion in the mean field approximation.

In order to consider the correlations, we have to go beyond the mean-field approximation and take into account so-called residual interaction, which is the difference between the bare electron–electron Coulomb repulsion

(6) $$\hat{w}=\frac{1}{2}\sum_{ijkl,s,s\prime }w_{ijkl}a_{i,s}^{†}a_{j,s\prime }^{†}a_{l,s\prime }a_{k,s},\;w_{ijkl}=\int \int d^{3}rd^{3}r^{\prime }\varphi_{i}^{*}\left(r\right)\varphi_{j}^{*}\left(r^{\prime }\right)\frac{1}{\left|r-r^{\prime }\right|}\varphi_{k}\left(r\right)\varphi_{l}\left(r^{\prime }\right)$$

and its part that was accounted for in the mean field [16]. The residual interaction is much more localized compared to the bare Coulomb interaction (6). That is why Hubbard proposed to use the basis of localized atomic-like Wannier functions and to take into account only the largest terms having i=j=k=l [21]. Then, the Hamiltonian ${\hat{H}}_{SCF}$ (1) is supplemented by the term

(7) $${\hat{H}}_{H,\mathrm{res}}=\hat{U}-{\hat{H}}_{c},\;\hat{U}=U\sum_{i}a_{i,↑}^{†}a_{i,↑}a_{i,↓}^{†}a_{i,↓},\;{\hat{H}}_{c}=U\left(\frac{n}{2}\sum_{i,s}a_{i,s}^{†}a_{i,s}-\frac{n^{2}}{4}\right),$$

where $n\equiv \langle g_{SCF}|\sum_{s}a_{i,s}^{†}a_{i,s}|g_{SCF}\rangle$. The one-particle Hamiltonian ${\hat{H}}_{c}$ subtracts the part of interaction $\hat{U}$, which was accounted for in the self-consistent field. As a result, the average $\langle g_{SCF}|{\hat{H}}_{H,\mathrm{res}}|g_{SCF}\rangle =0$. In our problem, n=1. The general form of ${\hat{H}}_{H,\mathrm{res}}$ for the full Coulomb interaction (6) is given in Eq. (2.3.35) of Ref. [16]. In our notations [the Coulomb matrix elements of Ref. [16] are connected with ours (6) via relation $V_{ikjl}=w_{ijkl}$], it is

(8) $${\hat{H}}_{\mathrm{res}}=\frac{1}{2}\sum_{ijkl,s,s\prime }w_{ijkl}a_{i,s}^{†}a_{j,s\prime }^{†}a_{l,s\prime }a_{k,s}-\sum_{ijkl,s}\left(w_{ikjl}-\frac{1}{2}w_{iljk}\right)P_{jl}a_{i,s}^{†}a_{k,s}+\frac{1}{2}\sum_{ijkl}\left(w_{ikjl}-\frac{1}{2}w_{iljk}\right)P_{ik}P_{jl},$$

where the bond order $P_{ij}\equiv \langle g_{SCF}|\sum_{s}a_{i,s}^{†}a_{j,s}|g_{SCF}\rangle$ is introduced.

Now, the Hamiltonian for the hydrogen molecule acquires the form of the Hubbard Hamiltonan

(9) $${\hat{H}}_{H}=\varepsilon \sum_{i,s}a_{i,s}^{†}a_{i,s}+\hat{t}+\hat{U},$$

where we have dropped the constant term $Un^{2}/4$. The diagonal matrix element is now $\varepsilon =\varepsilon_{SCF}-Un/2$. We take it as the zero of energy ε≡0. We shall solve the problem by the Heitler–London approach [22], which uses the many-body function basis. The Hamiltonian conserves the total spin *S* of the system. We can find the solution separately for singlet (S=0) and triplet (S=1) sectors of the Hilbert space. In addition, the Hamiltonian conserves the parity of the wave functions.

Triplet sector has three degenerate wave functions

(10) $$|t,+1\rangle =a_{1,↑}^{†}a_{2,↑}^{†}|vac\rangle ,\;|t,0\rangle =\frac{1}{\sqrt{2}}\left[a_{1,↑}^{†}a_{1,↓}^{†}-a_{2,↑}^{†}a_{2,↓}^{†}\right]|vac\rangle ,\;|t,-1\rangle =a_{1,↓}^{†}a_{2,↓}^{†}|vac\rangle .$$

The energy of the triplet is $E_{t}=2\varepsilon =0$.

In the singlet sector, we have three basis functions, one odd

(11) $$|s_{3}\rangle =\frac{1}{\sqrt{2}}\left[a_{1,↑}^{†}a_{1,↓}^{†}-a_{2,↑}^{†}a_{2,↓}^{†}\right]|vac\rangle$$

with the eigenenergy $E_{3}=U$, and two even: $|s_{1}\rangle$ and $|s_{2}\rangle$ (5). We obtain a 2×2 problem for even singlets

$${\hat{H}}_{H}|s_{1}\rangle =-2t|s_{2}\rangle ,\;{\hat{H}}_{H}|s_{2}\rangle =U|s_{2}\rangle -2t|s_{1}\rangle .$$

Its eigenvalues are

(12) $$E_{\nu }=\frac{U}{2}+\nu R,\;R\equiv \sqrt{{\left(\frac{U}{2}\right)}^{2}+4t^{2}}\approx \frac{U}{2}\left(1+\frac{8t^{2}}{U^{2}}\right),$$

where ν=±1. The last approximate equality (12) is valid in so-called strongly correlated limit U≫t. In this limit,

(13) $$E_{-}\approx -\frac{4t^{2}}{U},\;E_{+}\approx U+\frac{4t^{2}}{U}.$$

The eigenvectors are

(14) $$|g_{\nu }\rangle =\alpha_{\nu }|s_{1}\rangle +\beta_{\nu }|s_{2}\rangle ,\;\alpha_{\nu }=\frac{1}{\sqrt{2}}\sqrt{1-\frac{\nu U}{2R}},\;\beta_{\nu }=-\frac{\nu }{\sqrt{2}}\sqrt{1+\frac{\nu U}{2R}}.$$

Thus, for the ground state $|g_{S}\rangle =|g_{-}\rangle$, we have

(15) $$|g_{S}\rangle \approx \left(1-\frac{2t^{2}}{U^{2}}\right)|s_{1}\rangle +\frac{2t}{U}\left(1-\frac{6t^{2}}{U^{2}}\right)|s_{2}\rangle .$$

Comparing it with $|g_{SCF}\rangle$ (4), we see that the weight of ionic states $|s_{2}\rangle$ where electrons are found on the same site is strongly suppressed in the correlated wave function.

In the mean field approximation, the lowest excited state corresponds to one-electron excitation from bonding to antibonding orbital. It is separated from the ground state by the energy $\Delta E_{SCF}=2t$. In contrast to the mean field theory, the many-body approach obtains the first excited state of the system as *magnetic* excitation that flips the spin of one electron and transfers the singlet ground state $|g_{S}\rangle$ to one of the triplet states $|t,m\rangle$, m=0,±1. The excitation energy is

(16) $$\Delta E_{J}=E_{t}-E_{-}\approx \frac{4t^{2}}{U}=J\ll t.$$

The set of four lowest states $|g_{S}\rangle ,|t,m\rangle$ is separated from other states by an energy $\Delta E_{U}∼U$. It is easy to show that this set may be described by a low-energy effective Hamiltonian

(17) $${\hat{H}}_{J}=J{\hat{S}}_{1}\cdot {\hat{S}}_{2},$$

which is just the antiferromagnetic Heisenberg Hamiltonian.

#### Temperature Dependence of Optical Conductivity

**Figure 2:**
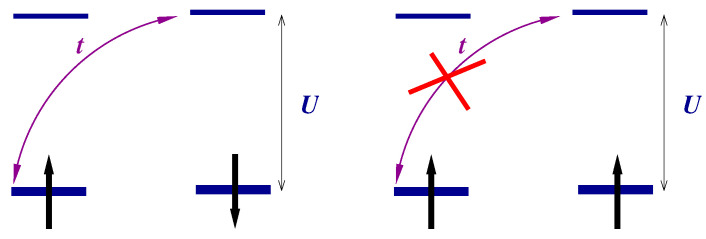
Description of the hydrogen molecule in the Heitler–London approach (account of correlations).

It is clear that the triplet states will be populated at temperatures $k_{B}T∼J$. The temperature dependence of some response functions of a strongly correlated system becomes observable at these temperatures. The values of effective exchange integral $J/k_{B}$ may vary from one to hundreds of Kelvins. Figure 2 illustrates the dependence of the charge response of the system on the magnetic initial state: the transition over the gap is allowed in the singlet state (left panel) and is prohibited in the triplet state (right panel) due to the Pauli exclusion principle. The population of the triplet states by temperature will evidently affect the response. Let us illustrate this statement by the calculation of the optical conductivity for an ensemble of molecules having a temperature *T*.

Optical conductivity for an ensemble of finite systems is given by (polarization of light is along the *x*-axis parallel to the molecule)

(18) $$\begin{array}{cc} \sigma_{xx}\left(z\right) & =\frac{1}{V}\langle \langle {\hat{P}}_{x}|{\hat{j}}_{x}\rangle \rangle =\frac{1}{Vz}\left[\langle \left[{\hat{P}}_{x},{\hat{j}}_{x}\right]\rangle +i\langle \langle {\hat{j}}_{x}|{\hat{j}}_{x}\rangle \rangle \right],\\ \langle \langle {\hat{j}}_{x}|{\hat{j}}_{x}\rangle \rangle & =\sum_{\mu }w_{\mu }\left(T\right){\left|\langle \mu |\hat{j}|\nu \rangle \right|}^{2}\left[\frac{1}{z-\omega_{\nu \mu }}-\frac{1}{z+\omega_{\nu \mu }}\right], \end{array}$$

where

(19) $$w_{\mu }\left(T\right)=\frac{\exp\left(-E_{\mu }/k_{B}T\right)}{\sum_{\mu^{\prime }}\exp\left(-E_{\mu^{\prime }}/k_{B}T\right)}$$

is the thermodynamic weight of the initial state $|\mu \rangle$, $\omega_{\nu \mu }=E_{\nu }-E_{\mu }$ is the energy of the transition, *V* is the volume per one system, ${\hat{P}}_{x}$ and ${\hat{j}}_{x}=-i\left[{\hat{P}}_{x},{\hat{H}}_{H}\right]$ are the polarization and the current operators.

The absorptive real part of the optical conductivity is

(20) Reσ(ω+i0,T)=∑μwμ(T)σμ(ω),

(21) $$\begin{array}{cc} \; & \sigma_{\mu }\left(\omega \right)=\frac{\pi }{V}\sum_{\nu }\frac{{\left|\langle \mu |\hat{j}|\nu \rangle \right|}^{2}}{\omega_{\nu \mu }}\left[\delta \left(\omega -\omega_{\nu \mu }\right)+\delta \left(\omega +\omega_{\nu \mu }\right)\right], \end{array}$$

where $\sigma_{\mu }\left(\omega \right)$ is the contribution to the optical conductivity of transitions from the state $|\mu \rangle$.

In our restricted basis for a two site system, only optical transitions with charge transfer between sites is possible. The current operator is

(22) $$\hat{j}=-ited\sum_{s}\left(a_{1,s}^{†}a_{2,s}-a_{2,s}^{†}a_{1,s}\right),$$

*d* is the distance between sites, and *e* is the electron charge. Non-zero matrix elements exist only between singlets $|s_{1}\rangle$ and $|s_{3}\rangle$. We thus have $\langle s_{1}|\hat{j}|s_{3}\rangle =-2ited$, and $\langle g_{\nu }|\hat{j}|s_{3}\rangle =-2ited\alpha_{\nu }$ and we are able to calculate the optical conductivity analytically for any temperature and parameters of Hamiltonian (9).

**Figure 3:**
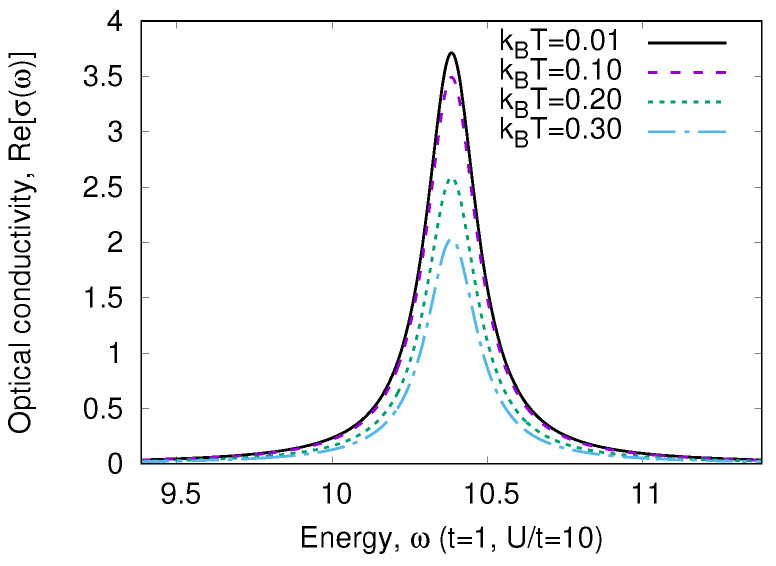
Optical conductivity Reσ(ω+i0,T) (20) for the model (9).

Figure 3 shows the optical conductivity for strongly correlated regime U/t=10 (a finite imaginary part η=0.1t was added to ω in order to visualize the δ-function). The typical value of *t* is ∼1 eV=11604 K$\cdot k_{B}$. Thus, for any realistic temperature, only the transition from the ground state (15) will be observed both in strongly (U≫t) and in weakly (U≪t) correlated limits. We may introduce the weight of the transition (the coefficient before $\delta \left(\omega -\omega_{\nu \mu }\right)$)

(23) $$W_{\nu \mu }\left(T\right)=\left[w_{\mu }\left(T\right)-w_{\nu }\left(T\right)\right]\frac{\pi {\left|\langle \mu |\hat{j}|\nu \rangle \right|}^{2}}{V\left(E_{\nu }-E_{\mu }\right)}.$$

**Figure 4:**
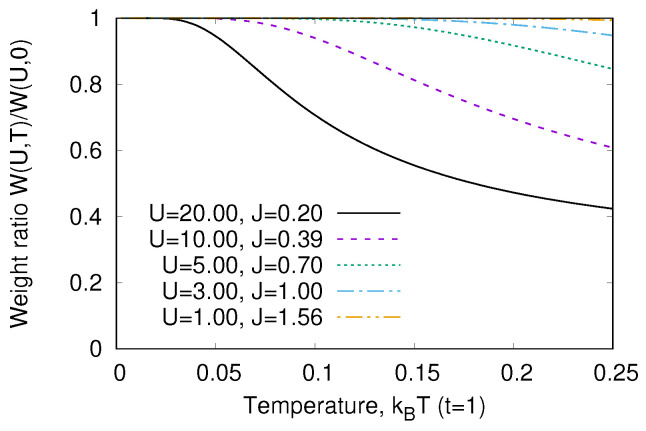
Optical weight $W_{3g}\left(T\right)/W_{3g}\left(0\right)$ (23) for various values of the Hamiltonian (9) parameters.

The temperature dependence of $W_{3g}$ is given in Figure 4. We see that the peculiarity of a strongly correlated system is a strong variation of W(T) with temperature. As the transition energy $\omega_{3g}∼U\gg T$, we may neglect $w_{3}\left(T\right)$. In addition, we may neglect all terms $∼\exp\left(-U/k_{B}T\right)$ in the denominator of Equation (19), which is the partition function. Then,

(24) $$w_{g}\left(T\right)\approx \frac{1}{1+3\exp\left[-\left(E_{t}-E_{-}\right)/k_{B}T\right]}=\frac{1}{1+3\exp\left(-J/k_{B}T\right)}.$$

This means that the characteristic temperature of optical response variation is the magnetic energy *J*. This is the general property of strongly correlated systems [23,24,25].

In the next subsection, we will show that the same conclusion holds for the temperature dependence of resonant inelastic X-ray scattering (RIXS) spectra for another simple finite system.

### 2.2. “Cu-O-Cu” Molecule. Temperature Dependence of RIXS Spectra

#### 2.2.1. The Hamiltonian and Its Spectrum

**Figure 5:**
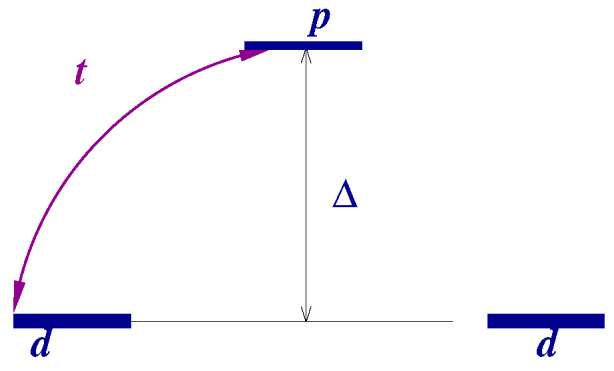
Energy level scheme in a “Cu-O-Cu” molecule.

We consider the three-site “Cu-O-Cu” cluster, which is described by a many-band Hubbard model (see Figure 5) [26]

(25) $$\hat{H}=\Delta \sum_{\sigma }p_{\sigma }^{†}p_{\sigma }+t\sum_{i,\sigma }\left(Z_{i}^{\sigma 0}p_{\sigma }+p_{\sigma }^{†}Z_{i}^{0\sigma }\right),$$

where the operator $p_{\sigma }^{†}$ creates a particle with the spin projection index σ=↑,↓ on the uncorrelated “O” site,

(26) $$Z_{i}^{\sigma 0}\equiv |i,\sigma \rangle \langle i,0|=d_{\sigma }^{†}\left(1-d_{-\sigma }^{†}d_{-\sigma }\right)$$

is the Hubbard projection operator that creates a particle with the spin projection index σ on the site i=1,2, where strong correlations prohibit the double occupancy of the site (see Appendix C). Thus, we consider a limiting case of the Emery (multi-band Hubbard) model [27] (later in Section 4.2 we will discuss the Emery model for cuprates more in detail) when $1/U_{d}=U_{p}=0$, $U_{d}$($U_{p}$) being on-site repulsion on the “Cu”(“O”) site. The remaining energetic parameters are the charge-transfer energy Δ (positive for hole representation), and the hopping *t*. The typical values for cuprates are Δ∼4, t∼1 eV, and below we give the analytic formulas for exact values together with the expansion over t/Δ≪1. We consider the insulating case when we have two holes in the cluster.

**Figure 6:**
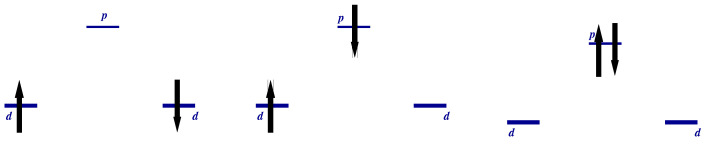
Scheme of $|s_{d}\rangle$, $|ZRS1\rangle$, and $|s_{p}\rangle$ states (27)–(30) in a “Cu-O-Cu” molecule.

*The Classification of States.* We may characterize the wave function by total spin value, its projection on the *z*-axis, and parity with respect to exchange of “Cu” sites 1↔2. In the two-particle sector, we have (Figure 6)

(i) S=0

(1) even:

(27) $$\begin{array}{cc} |s_{d}\rangle & =\frac{1}{\sqrt{2}}\left(Z_{1}^{↑0}Z_{2}^{↓0}-Z_{1}^{↓0}Z_{2}^{↑0}\right)|vac\rangle , \end{array}$$

(28) $$\begin{array}{cc} |ZRSs\rangle & =\frac{1}{\sqrt{2}}\left[|ZRS,1\rangle +|ZRS,2\rangle \right], \end{array}$$

(29) $$\begin{array}{cc} |s_{p}\rangle & =p_{↑}^{†}p_{↓}^{†}|vac\rangle , \end{array}$$

where

(30) $$|ZRS,i\rangle =\frac{1}{\sqrt{2}}\left(Z_{i}^{↑0}p_{↓}^{†}-Z_{i}^{↓0}p_{↑}^{†}\right)|vac\rangle ,$$

is the Zhang–Rice singlet state [28,29] formed by holes on neighboring Cu-O sites.

(2) odd:

(31) $$|ZRSa\rangle =\frac{1}{\sqrt{2}}\left[|ZRS,1\rangle -|ZRS,2\rangle \right],$$

(ii)S=1, $S^{z}=1$

(1) even:

(32) $$|ts\rangle =\frac{1}{\sqrt{2}}p_{↑}^{†}\left(Z_{1}^{↑0}+Z_{2}^{↑0}\right)|vac\rangle ,$$

(2) odd (Figure 7):

(33) $$\begin{array}{cc} |t_{d}\rangle & =Z_{1}^{↑0}Z_{2}^{↑0}|vac\rangle , \end{array}$$

(34) $$\begin{array}{cc} |ZRT\rangle & =\frac{1}{\sqrt{2}}p_{↑}^{†}\left(Z_{2}^{↑0}-Z_{1}^{↑0}\right)|vac\rangle . \end{array}$$

The wave functions with other values of $S^{z}$ may be obtained by the action of operator ${\hat{S}}^{-}$ on the above states.

*Summary of the spectrum.* The Hamiltonian (25) has 13 two-particle eigenstates (See Appendix A.1 for the details): 4 singlets (31), (A6), and 3 triplets (32), (A12), and (A13). In the low-energy states $|f=0\rangle$(A6) and $|Gt\rangle$ (A12), the dominant contribution comes from the states $|s_{d}\rangle$(27) and $|t_{d}\rangle$(33), both having two particles occupying different “Cu” sites. In this low-energy part of the spectrum, the system has only spin degrees of freedom which are described by an effective Heisenberg Hamiltonian

(35) $$\hat{J}=J{\hat{S}}_{1}\cdot {\hat{S}}_{2}+2e_{d}$$

with the superexchange parameter $J=E_{t,0}-E_{0}\approx 4t^{4}/\Delta^{3}\ll t,\Delta$. The constant shift $2e_{d}=-2t^{2}/\Delta$ comes from the hybridization contribution to the “crystal field” on the “Cu” site.

The excited states corresponding to a transfer of charge between the “Cu” and the “O” sites lie higher in energy by the value about Δ.

#### 2.2.2. O *K* RIXS Spectrum for Finite Temperature

*General Expression* The O *K* RIXS process has the following stages: (i) in a system being in an initial state $|g\rangle$, an X-ray quantum excites an electron from an oxygen core 1s-state to a 2p-state on the same site R; (ii) the valence electron system propagates in the presence of *the immovable core-hole* at site R; (iii) the 2p-electron recombines *on the same site* R with the core 1s-hole, another X-ray quantum is emitted, and the system is left in a finite state $|f\rangle$.

The RIXS spectrum intensity at finite temperature is given by (see e.g., [30,31,32,33])

(36) $$I\left(T,\Omega ,\omega ,\vec{\epsilon },{\vec{\epsilon }}^{\prime }\right)={\langle I_{g}\left(\Omega ,\omega ,\vec{\epsilon },{\vec{\epsilon }}^{\prime }\right)\rangle }_{T}=\frac{\sum_{g}\exp\left(-E_{g}/kT\right)I_{g}\left(\Omega ,\omega ,\vec{\epsilon },{\vec{\epsilon }}^{\prime }\right)}{\sum_{g}\exp\left(-E_{g}/kT\right)},$$

where ${\langle \cdots \rangle }_{T}$ denote the statistical average over initial states $|g\rangle$ for a temperature *T*, k≈1/11604 eV/K is the Boltzmann constant, $\vec{\epsilon }$(${\vec{\epsilon }}^{\prime }$) is the polarization vectors of incident(emitted) photons, and Ω and ω are the incident and emitted photon energies.

For a given $|g\rangle$, the intensity of the O *K* RIXS signal is (see e.g., [31,32,33])

(37) $$I_{g}\left(\Omega ,\omega ,\vec{\epsilon },{\vec{\epsilon }}^{\prime }\right)=\sum_{f}{\left|\sum_{R,m,\mu ,\nu }\frac{\langle f|\epsilon_{\mu }^{\prime }{\hat{T}}_{\mu ,R}|m,R\rangle \langle m,R|\epsilon_{\nu }{\hat{T}}_{\nu ,R}|g\rangle }{E_{g}+\Omega -E_{m,R}-ı\Gamma }\right|}^{2}\delta \left(E_{g}+\Omega -E_{f}-\omega \right)$$

where ${\hat{T}}_{\mu ,R}=\sum_{\sigma }s_{R\sigma }^{†}p_{R\mu \sigma }+h.c.$, $s_{R\sigma }^{†}$ is the creation operator of the O 1s-hole with spin projection σ at site R, $p_{R\mu \sigma }^{†}$ creates a 2p-hole at the same site, μ and ν are Cartesian indices of 2p-orbitals, and $|m,R\rangle$ is the eigenstate of the Hamiltonian of the system in the presence of the core hole at site R (in the stage ii)

(38) $${\hat{H}}_{R}={\hat{H}}_{pd}+{\hat{H}}_{C,R},\;{\hat{H}}_{C,R}=\varepsilon_{s}\sum_{\sigma }s_{R\sigma }^{†}s_{R\sigma }+Q\sum_{\sigma ,\sigma^{\prime },\mu }s_{R\sigma }^{†}s_{R\sigma }p_{R\mu \sigma^{\prime }}^{†}p_{R\mu \sigma^{\prime }}.$$

In Equation (38), the first term ${\hat{H}}_{pd}$ is the generalized many-band Hubbard Hamiltonian that describes the valence electron system of cuprates; ${\hat{H}}_{C,R}$ describes the O 1s hole and its interaction with valence *p*-holes which is assumed to be reduced to local Coulomb repulsion. The sum in Equation (37) runs over sites R where the core hole is created at stage (i), rests at stage (ii), and annihilates at stage (iii), cf. Equation (3) of Ref. [33] where apparently a triple sum over R is present. In fact, the expression (38) implies that the core hole does not move and is annihilated at the same site where it was created. This reduces the triple sum to a single one.

With the assumptions inherent in Equation (38), the role of the core hole is reduced to the change of the on-site energy of valence *p*-states at the stage (ii) of the RIXS process. This may be shown in the following way: let us recall that the core hole is absent in the initial and final states, i.e., we can write $|f\rangle =|f\rangle ⊗|0_{C}\rangle$, $|0_{C}\rangle$ being the vacuum for core states. Then, we have

(39) $$\begin{array}{cc} \; & \frac{\langle 0_{C}|{\hat{T}}_{\mu ,R}|m,R\rangle \langle m,R|{\hat{T}}_{\nu ,R}|0_{C}\rangle }{E_{g}+\Omega -E_{m,R}-ı\Gamma }=\langle 0_{C}|\sum_{\gamma ,\sigma }p_{R\mu \gamma }^{†}s_{R\gamma }\frac{1}{z-\hat{H_{pd}}-{\hat{H}}_{C,R}}s_{R\sigma }^{†}p_{R\nu \sigma }|0_{C}\rangle \\ \; & \langle 0_{C}|s_{R\gamma }\frac{1}{z-{\hat{H}}_{pd}-{\hat{H}}_{C,R}}s_{R\sigma }^{†}|0_{C}\rangle =\langle 0_{C}|s_{R\gamma }\frac{1}{z-\hat{H_{pd}}}\left[\sum_{n=0}^{\infty }{\left({\hat{H}}_{C,R}\frac{1}{z-{\hat{H}}_{pd}}\right)}^{n}\right]s_{R\sigma }^{†}|0_{C}\rangle \\ \; & =\langle 0_{C}|\frac{1}{z-{\hat{H}}_{pd}}\sum_{n=0}^{\infty }{\left[\left(\varepsilon_{s}+Q\sum_{\sigma^{\prime },\alpha }p_{R\alpha \sigma^{\prime }}^{†}p_{R\alpha \sigma^{\prime }}\right)\frac{1}{z-{\hat{H}}_{pd}}\right]}^{n}s_{R\gamma }s_{R\sigma }^{†}|0_{C}\rangle \\ \; & =\frac{1}{z-{\hat{H}}_{pd}-\left(\varepsilon_{s}+Q\sum_{\sigma^{\prime },\alpha }p_{R\alpha \sigma^{\prime }}^{†}p_{R\alpha \sigma^{\prime }}\right)}\delta_{\gamma \sigma }, \end{array}$$

with $z\equiv E_{g}+\Omega -ı\Gamma$. In the derivation of (39), we have used the relation

(40) $$\langle 0_{C}|s_{R\gamma }{\hat{H}}_{C,R}=\langle 0_{C}|\left(\left[s_{R\gamma },{\hat{H}}_{C,R}\right]+{\hat{H}}_{C,R}s_{R\gamma }\right)=\langle 0_{C}|\left(\varepsilon_{s}+Q\sum_{\sigma^{\prime },\mu }p_{R\mu \sigma^{\prime }}^{†}p_{R\mu \sigma^{\prime }}\right)s_{R\gamma }.$$

The substitution of (39) into (37) gives Equation (41). We see that the O *K* RIXS spectral function (within the approximation made) is defined by the dynamics of valence electrons only. Thus, we obtain

(41) $$\begin{array}{cc} \; & I_{g}\left(\Omega ,\omega ,\vec{\epsilon },{\vec{\epsilon }}^{\prime }\right)=\sum_{f}{\left|\sum_{R,\mu ,\nu }\epsilon_{\mu }^{\prime }\epsilon_{\nu }M_{R\mu \nu }^{fg}\left(\Omega \right)\right|}^{2}\delta \left(E_{g}+\Omega -E_{f}-\omega \right), \end{array}$$

(42) $$\begin{array}{cc} \; & M_{R\mu \nu }^{fg}\left(\Omega \right)=\sum_{\sigma }\langle f|p_{R\mu \sigma }^{†}\frac{1}{z-\hat{H_{pd}}-\left(\varepsilon_{s}+U_{C}\sum_{\sigma^{\prime },\alpha }p_{R\alpha \sigma^{\prime }}^{†}p_{R\alpha \sigma^{\prime }}\right)}p_{R\nu \sigma }|g\rangle , \end{array}$$

where $p_{R\mu \sigma }^{†}$ creates a 2p-hole with spin projection σ at oxygen site R, μ=x,y,z. ${\hat{H}}_{pd}$ is the generalized many-band Hubbard Hamiltonian that describes the valence electron system, $\varepsilon_{s}$ is the energy of 1s hole level, and $U_{C}$ is the interaction strength between the 1s- and valence 2p-holes. The interaction is assumed to be reduced to local Coulomb repulsion.

The expressions (36)–(42) involves only valence states. The stages (i)–(iii) may be reformulated as:

(i${}^{\prime }$) in a system being in the *N*-hole ground state $|g\rangle$, a hole in a 2p-state on the site R is annihilated;

(ii${}^{\prime }$) the N−1 hole system is perturbed by the increase of site energy on the site R by the value $U_{C}$;

(iii${}^{\prime }$) the 2p−hole on the same site R is created, and the system is left in an excited state $|f\rangle$.

*Application to the Three-Site Model* As we have already mentioned, the charge-transfer excitations have energies of the order of several eV. They will never be populated at temperatures reachable in an RIXS experiment (T<0.1 eV). So, we should make the statistical average only over the low-energy states

$$I\left(T,\Omega ,\omega \right)=w_{s}I_{0}\left(\Omega ,\omega \right)+w_{t}I_{t}\left(\Omega ,\omega \right),$$

where $w_{s}=1/Q\left(T\right)$ and $w_{t}=3\exp\left(-J/kT\right)/Q\left(T\right)$ are statistical weights of the lowest singlet and triplet states, and Q(T)=1+3exp(−J/kT) is the partition function.

In the intermediate state, our system has only one hole. The eigenenergies of even states are

(43) $$E_{\pm }=\frac{\Delta +U_{C}}{2}\pm r,$$

where $r=\sqrt{1+8\left[t/\left(\Delta +U_{C}\right)\right]{}^{2}}$. The eigenstates are

(44) $$\begin{array}{cc} |\sigma ,+\rangle & =\cos\gamma p_{\sigma }^{†}|vac\rangle +\sin\gamma \frac{1}{\sqrt{2}}\left(Z_{1}^{\sigma 0}+Z_{2}^{\sigma 0}\right)|vac\rangle , \end{array}$$

(45) $$\begin{array}{cc} |\sigma ,-\rangle & =\sin\gamma |t_{d}\rangle -\cos\gamma \frac{1}{\sqrt{2}}\left(Z_{1}^{\sigma 0}+Z_{2}^{\sigma 0}\right)|vac\rangle , \end{array}$$

here sinγ and cosγ are given by expressions similar to Equations (A14) with the change $R_{t}\to r$, and $\Delta \to \Delta +U_{C}$.

**Figure 8:**
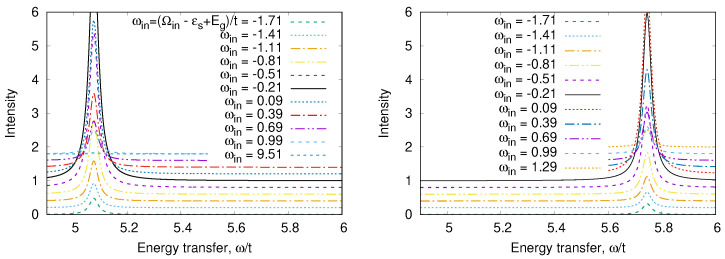
RIXS for $|s_{d}\rangle$ (left) and $|t_{d}\rangle$ (right) starting states in a “Cu-O-Cu” molecule.

**Figure 9:**
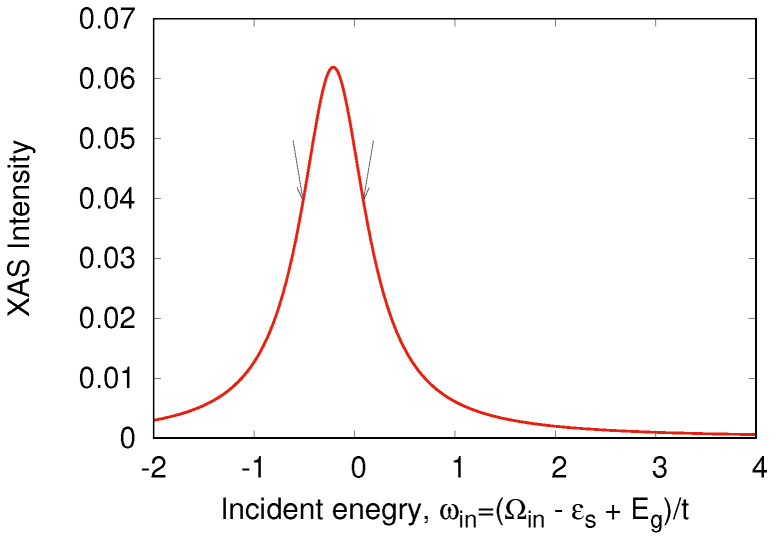
XAS spectrum in a “Cu-O-Cu” molecule. Arrows show the input X-ray frequencies.

**Figure 10:**
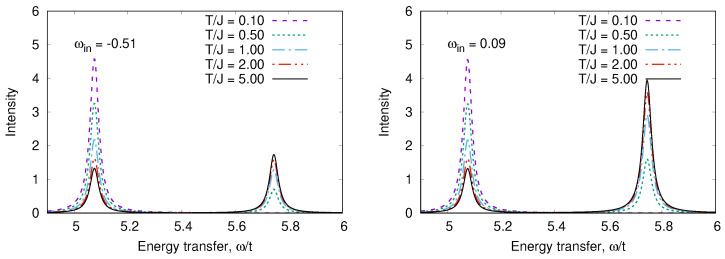
*T*-dependence of RIXS spectra in a “Cu-O-Cu” molecule for two input frequencies.

**Figure 11:**
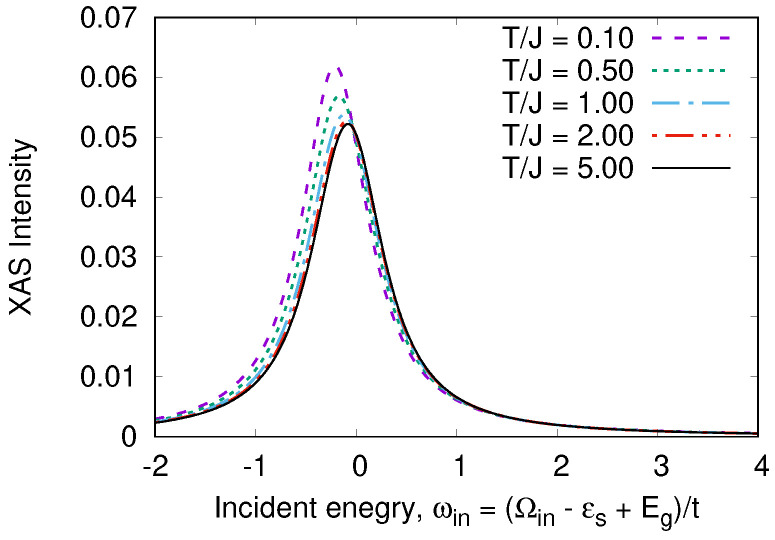
*T*-dependence of XAS spectrum in a “Cu-O-Cu” molecule.

An odd state $|\sigma ,a\rangle =\frac{1}{\sqrt{2}}\left(Z_{1}^{\sigma 0}-Z_{2}^{\sigma 0}\right)|vac\rangle$ has the energy $E_{a}=0.$ The calculated RIXS and XAS spectra are shown in Figure 8, Figure 9, Figure 10 and Figure 11. We clearly see the resonant character of the spectra shown on Figure 8. The resonsnce occurs at different incident energies for singlet and triplet initial states. The occupation of different initial states depends on temperature. This leads to temperature dependence of the RIXS spectra. The changes of temperature on the scale of magnetic interaction value *J* leads to drastic changes of spectra on a much larger scale t≫J as shown in Figure 10.

The strong temperature dependence of the RIXS spectrum was first observed for ${\mathrm{Li}}_{2}{\mathrm{CuO}}_{2}$ and ${\mathrm{CuGeO}}_{3}$ edge-shared cuprate compounds in Ref. [34]

The XAS spectrum also depends on temperature, but this dependence is weak.

## 3. Effective Low-Energy Models

### 3.1. Resolvent Method (Löwdin Downfolding)

This method provides the simplest way to obtain a low-energy effective Hamiltonian from a full Hamiltonian of a system. It was proposed in a series of works of P.-O. Löwdin (e.g. Refs. [35,36]), where he called this method the “Partitioning technique”. Here, we briefly review the technique.

We assume that a full Hilbert space of states of a system described by a Hamiltonian $\hat{H}$ may be divided into two parts *A* and *B*, *A* being an “interesting low-energy part” in some sense. Then, we may write the Hamiltonian matrix and an eigenvector symbolically

(46) $$H=\left(\begin{array}{cc} H_{A} & H_{AB}\\ H_{AB}^{†} & H_{B} \end{array}\right),\;c=\left(\begin{array}{c} a\\ b \end{array}\right).$$

Then, the secular equation Hc=Ec becomes

(47) $$\left\{\begin{array}{ccc} H_{A}a+H_{AB}b & = & Ea,\\ H_{AB}^{†}a+H_{B}b & = & Eb. \end{array}\right.$$

We substitute the expression for *b* found from the second equation

(48) $$b=\frac{1}{E-H_{B}}H_{AB}^{†}a$$

into the first one and obtain

(49) $$\left(H_{A}+H_{AB}\frac{1}{E-H_{B}}H_{AB}^{†}\right)a=Ea.$$

Equation (49) looks like an effective secular equation $H_{eff}a=Ea$ for a matrix in the subspace *A*, but $H_{eff}$ contains the inversion of a large matrix $E-H_{B}$. This inversion is calculated iteratively. We separate $H_{B}$ into diagonal and non-diagonal parts $H_{B}=H_{0,B}+V_{B}$ and expand ${\left(E-H_{0,B}-V_{B}\right)}^{-1}$ in a power series in $V_{B}$. Then, we have up to fourth order

(50) $$\begin{array}{cc} H_{eff}\left(E\right) & =H_{A}+H_{AB}\frac{1}{E-H_{0,B}}H_{AB}^{†}+H_{AB}\frac{1}{E-H_{0,B}}V_{B}\frac{1}{E-H_{0,B}}H_{AB}^{†}\\ \; & +H_{AB}\frac{1}{E-H_{0,B}}V_{B}\frac{1}{E-H_{0,B}}V_{B}\frac{1}{E-H_{0,B}}H_{AB}^{†}+\cdots . \end{array}$$

Note that $H_{eff}\left(E\right)$ depends on the eigenenergy. So, Equation (49) is a non-linear equation. We should write $E=E^{\left(0\right)}+E^{\left(2\right)}+\cdots$, and solve the equation iteratively.

Let us pay attention to an outstanding feature of the expansion (50). It never diverges if the states in the subspace *A* are separated by an energy gap from the states in *B*. This is the case when we derive an effective magnetic Hamiltonian for a strongly correlated insulating system. Then, the subspace *B* contains the states with charge excitations which are separated by a charge-transfer or a Hubbard gap for the charge-transfer or Mott–Hubbard insulators, respectively (for the classification of correlated systems see Section 4.4).

### 3.2. The Effective Hamiltonian after Fourth-Order Canonical Transform

Let us consider another way to obtain an effective Hamiltonian. We denote

(51) $$\hat{H}={\hat{H}}_{0}+\hat{V},\;{\hat{H}}_{0}=\sum_{m}|m\rangle E_{m}\langle m|,\;\hat{V}=\sum_{m\neq n}|n\rangle t_{nm}\langle m|,$$

where the eigenvalues $E_{m}$ and eigenvectors $|m\rangle$ of an unperturbed Hamiltonian ${\hat{H}}_{0}$ are assumed to be known. Without loss of generality, we may assume that the perturbation $\hat{V}$ contains only non-diagonal terms. The operator

(52) $$\hat{W}=\sum_{n\neq m}|n\rangle \frac{t_{nm}}{E_{m}-E_{n}}\langle m|$$

has the property

(53) $$\left[{\hat{H}}_{0},\hat{W}\right]\equiv {\hat{H}}_{0}\hat{W}-\hat{W}{\hat{H}}_{0}=-\hat{V}.$$

Then, up to the fourth order, the canonical transformation gives

(54) $$\begin{array}{cc} {\hat{H}}_{eff} & =\exp\left(-\hat{W}\right)\hat{H}\exp\left(\hat{W}\right)={\hat{H}}_{0}+{\hat{H}}_{2}+{\hat{H}}_{3}+{\hat{H}}_{41}\\ \; & ={\hat{H}}_{0}+\frac{1}{2}\left[\hat{V},\hat{W}\right]+\frac{1}{3}\left[\left[\hat{V},\hat{W}\right],\hat{W}\right]+\frac{1}{8}\left[\left[\left[\hat{V},\hat{W}\right],\hat{W}\right],\hat{W}\right]. \end{array}$$

The explicit calculation gives

(55) $$\begin{array}{cc} {\hat{H}}_{2} & =\frac{1}{2}\sum_{n,m,j}|n\rangle t_{nj}t_{jm}D_{njm}\langle m|,\;{\hat{H}}_{31}=\frac{1}{3}\sum_{n,j,k,m}|n\rangle t_{nj}t_{jk}t_{km}D_{njkm}\langle m|, \end{array}$$

(56) $$\begin{array}{cc} {\hat{H}}_{41} & =\frac{1}{8}\sum_{n,j,k,l,m}|n\rangle t_{nj}t_{jk}t_{kl}t_{lm}D_{njklm}\langle m|, \end{array}$$

(57) $$\begin{array}{cc} D_{njm} & \equiv \left(\frac{1}{E_{nj}}-\frac{1}{E_{jm}}\right),\;E_{mj}\equiv E_{m}-E_{j}, \end{array}$$

(58) $$\begin{array}{cc} D_{njkm} & \equiv \frac{1}{E_{nj}}D_{jkm}-D_{njk}\frac{1}{E_{km}}=\frac{1}{E_{nj}E_{jk}}-\frac{2}{E_{nj}E_{km}}+\frac{1}{E_{jk}E_{km}}, \end{array}$$

(59) $$\begin{array}{cc} D_{njklm} & \equiv \frac{1}{E_{nj}}D_{jklm}-D_{njkl}\frac{1}{E_{lm}}=\frac{1}{E_{nj}E_{jk}E_{kl}}+\frac{3}{E_{nj}E_{jk}E_{ml}}-\frac{3}{E_{nj}E_{kl}E_{ml}}+\frac{1}{E_{jk}E_{kl}E_{ml}}. \end{array}$$

The advantage of this approach is that it gives an energy-independent effective Hamiltonian. However, we can see a substantial difference of the Hamiltonian (54) from (50). The denominators of the third and fourth orders (58) and (59) contain the energy differences between intermediate states. These terms may diverge if the states are (quasi-)degenerate even if these states are well separated by an energy gap from the states in the subspace A. In fact, the generator $\hat{W}$ has excluded the non-diagonal terms due to the property (53) only up to the second order. For the derivation of the fourth order effective Hamiltonian, we need to also exclude non-diagonal terms in ${\hat{H}}_{2}$. So, we perform a second transform with the generator

(60) $$\begin{array}{cc} {\hat{W}}_{2} & =\sum_{n\neq m}|n\rangle \frac{\theta_{nm}}{E_{m}-E_{n}}\langle m|,\;\theta_{nm}\equiv \frac{1}{2}\sum_{j}|n\rangle t_{nj}t_{jm}D_{njm}\langle m|. \end{array}$$

Then, the fourth-order term becomes

(61) $$\begin{array}{cc} {\hat{H}}_{4} & ={\hat{H}}_{41}+{\hat{H}}_{42}=\frac{1}{8}\sum_{n,j,k,l,m}|n\rangle t_{nj}t_{jk}t_{kl}t_{lm}G_{njklm}\langle m|, \end{array}$$

(62) $$\begin{array}{cc} {\hat{H}}_{42} & =\frac{1}{2}\sum_{n,m,k}|n\rangle \theta_{nk}\theta_{km}D_{nkm}\langle m|=\frac{1}{8}\sum_{n,m,j}|n\rangle t_{nj}t_{jk}D_{njk}t_{kl}t_{lm}D_{klm}D_{nkm}\langle m|, \end{array}$$

(63) $$\begin{array}{cc} G_{njklm} & =D_{njk}D_{klm}D_{nkm}+D_{njklm}. \end{array}$$

After some algebra (see Appendix B for the details), we obtain

(64) $$\begin{array}{cc} G_{njklm} & =\frac{4}{E_{nj}E_{ml}}\left(\frac{1}{E_{mk}}+\frac{1}{E_{nk}}\right)+\frac{2E_{mn}}{E_{nj}E_{ml}}\left(\frac{1}{E_{mk}E_{jk}}+\frac{1}{E_{nk}E_{kl}}\right). \end{array}$$

Now, compare Equation (61) with $G_{njklm}$ given by (64) and the Löwdin result (50). We see that the first term of (64) produces the form similar to (50), if we assume that both $|m\rangle ,|n\rangle$ belong to the subspace *A*. The second term of (64) looks different. However, we can note the following: (i) it vanishes when $E_{m}=E_{n}$, i.e., when the subspace *A* is degenerate; (ii) we have written the transformation that tries to remove *all* non-diagonal terms in (51), whereas only the part of them, namely $H_{AB}$, are removed in the Löwdin approach. If we divide $\hat{V}$ in (51), into

(65) $$\hat{V}={\hat{V}}_{AB}+{\hat{V}}_{A}+{\hat{V}}_{B}$$

and make the transformation that removes only ${\hat{V}}_{AB}$, we will have the form similar to (50), but in practice, it is difficult to make the decomposition (65).

As we mentioned above, one of the advantage of the canonical transform is that it gives an energy-independent effective Hamiltonian. The second important advantage is that it easily gives the transformed form of any operator to the same order:

(66) $$\tilde{A}=\exp\left(-{\hat{W}}_{2}\right)\exp\left(-\hat{W}\right)\hat{A}\exp\left(\hat{W}\right)\exp\left({\hat{W}}_{2}\right),$$

the transformed form of the wave function may be found as well.

## 4. Models for Electronic Structure of Strongly Correlated Systems

### 4.1. Hubbard Model

One of the simplest models that shows the peculiarities of the physics of strongly correlated electron systems was introduced by J. Hubbard in Ref. [21] (implicitly this model was used by P.W. Anderson for consideration of the superexchange [37]). The Hubbard Hamiltonian reads

(67) $$\hat{H}=\hat{t}+\hat{U},\;\hat{t}=-t\sum_{R,\sigma }a_{R,\sigma }^{†}\left(\sum_{g}a_{R+g,\sigma }\right),\;\hat{U}=U\sum_{R}{\hat{n}}_{R↑}{\hat{n}}_{R↓},$$

where the summation goes over the sites R of a lattice (here we consider an infinite crystal lattice), $a_{R,\sigma }^{†}$ creates an electron in a state with a wave function ϕ(r−R) localized at a site R with spin projection σ, ${\hat{n}}_{R,\sigma }=a_{R,\sigma }^{†}a_{R,\sigma }$ is the on-site operator of the number of electrons with spin projection σ, and vector g joins nearest neighbors.

In the mean field approximation, the Hamiltonian reduces to a single-band tight-binding Hamiltonian

(68) $${\hat{H}}_{SCF}=\sum_{k}\left(t_{k}+U\bar{n}\right)a_{k,\sigma }^{†}a_{k,\sigma },\;t_{k}=-t\sum_{g}e^{i\mathrm{kg}},$$

where the operator annihilating an electron in a band state is given by the Fourier transform

(69) $$a_{k,\sigma }=\frac{1}{\sqrt{N}}\sum_{R}e^{-ikR}a_{R,\sigma },$$

where *N* is the number of sites in the lattice. Our system is translationally invariant; thus, the on-site average of the electron number $\langle \sum_{\sigma }{\hat{n}}_{R,\sigma }\rangle =n$ does not depend on R. Here, we consider a non-spin-polarized case, when also $\langle {\hat{n}}_{R,\sigma }\rangle =\bar{n}=n/2$ does not depend on σ and R.

The simplest approximation that shows the strongly correlated behavior for

(70) U≫t

is the so-called Hubbard-I approximation [21], which is a decoupling scheme for the two-time Green’s function technique.

The aim is the calculation of the retarded Green’s function

(71) $$G_{\sigma }\left(r,r^{\prime },\omega \right)=\langle \langle \psi_{\sigma }\left(r\right)|\psi_{\sigma }^{†}\left(r^{\prime }\right)\rangle \rangle =-i\int_{0}^{\infty }\langle \left\{\psi_{\sigma }\left(r,t\right),\psi_{\sigma }^{†}\left(r^{\prime },0\right)\right\}\rangle e^{ı\omega t}dt,$$

where ${\left[A,B\right]}_{\eta }\equiv AB-\eta BA$, the time dependence of an operator $\hat{A}\left(t\right)$ is given by $\hat{A}\left(t\right)=e^{it\hat{H}}\hat{A}e^{-it\hat{H}}$, and the angular brackets denote the thermodynamic average

(72) $$\langle \hat{A}\rangle \equiv Tr\left[\exp\left(-\beta \hat{H}\right)\hat{A}\right]/Tr\left[\exp\left(-\beta \hat{H}\right)\right],$$

operator

(73) $$\psi_{\sigma }^{†}\left(r\right)=\sum_{R}\phi^{*}\left(r-R\right)a_{R,\sigma }^{†}$$

creates an electron with spin projection σ at point r;

(74) $$G_{\sigma }\left(r,r^{\prime },\omega \right)=\sum_{R,R^{\prime }}\phi \left(r-R\right)\phi^{*}\left(r^{\prime }-R^{\prime }\right)G_{\sigma ,R,R^{\prime }}\left(\omega \right),\;G_{\sigma ,R,R^{\prime }}\left(\omega \right)=\langle \langle a_{R,\sigma }|a_{R^{\prime },\sigma }^{†}\rangle \rangle .$$

We consider the system in a non-magnetic state and will drop the spin index of the Green’s function. The equation of motion for the Green’s function reads

(75) $$\begin{array}{cc} \omega G_{R,R^{\prime }} & =\delta_{R,R^{\prime }}-t\sum_{g}G_{R+g,R^{\prime }}+U\Gamma_{R,R^{\prime }}, \end{array}$$

(76) $$\begin{array}{cc} \Gamma_{R,R^{\prime }} & \equiv \langle \langle a_{R.\sigma }{\hat{n}}_{R,-\sigma }|a_{R^{\prime },\sigma }^{†}\rangle \rangle , \end{array}$$

The Hubbard-I decoupling is introduced in the equation for higher-order function (76)

(77) $$\begin{array}{cc} \; & \left(\omega -U\right)\Gamma_{R,R^{\prime }}=\langle {\hat{n}}_{R,\sigma }\rangle \delta_{RR^{\prime }}-t\langle \langle c_{R,\sigma }|a_{R^{\prime },\sigma }^{†}\rangle \rangle ,\\ \; & c_{R,\sigma }\equiv \sum_{g}a_{R+g,\sigma }{\hat{n}}_{R,-\sigma }+a_{R,\sigma }a_{R,-\sigma }^{†}\sum_{g}a_{R+g,-\sigma }-a_{R,\sigma }\left(\sum_{g}a_{R+g,-\sigma }^{†}\right)a_{R,-\sigma },\\ \; & \langle \langle c_{R,\sigma }|a_{R^{\prime },\sigma }^{†}\rangle \rangle ≃\langle {\hat{n}}_{R,-\sigma }\rangle \sum_{g}G_{R+g,R^{\prime }} \end{array}$$

In a non-magnetic state, the average does not depend on σ. Then, Equation (77) becomes

(78) $$\Gamma_{R,R^{\prime }}=\bar{n}\frac{\delta_{RR^{\prime }}-t\sum_{g}G_{R+g,R^{\prime }}}{\omega -U},$$

and we find

(79) $$\begin{array}{cc} \omega G_{R,R^{\prime }} & =\left(1+\frac{U\bar{n}}{\omega -U}\right)\left[\delta_{RR^{\prime }}-t\sum_{g}G_{R+g,R^{\prime }}\right]. \end{array}$$

This equation may be solved using Fourier transform

(80) $$\begin{array}{cc} \; & \omega G_{k,k^{\prime }}\equiv \frac{\omega }{N}\sum_{R,R^{\prime }}\exp\left(-i\mathrm{kR}+ik^{\prime }R^{\prime }\right)G_{R,R^{\prime }}\\ \; & =\frac{\omega }{N}\sum_{R,R^{\prime }}\exp\left(-i\mathrm{kR}+ik^{\prime }R^{\prime }\right)\left\{\left(1+\frac{U\bar{n}}{\omega -U}\right)\left[\delta_{RR^{\prime }}-t\sum_{g}G_{R+g,R^{\prime }}\right]\right\}\\ \; & =Z\left(\omega \right)\left(\delta_{kk^{\prime }}+t_{k}G_{k,k^{\prime }}\right), \end{array}$$

where

(81) $$Z\left(\omega \right)\equiv 1+\frac{U\bar{n}}{\omega -U},$$

From the last Equation (80), we obtain

(82) $$G_{k,k^{\prime }}=\delta_{kk^{\prime }}G_{k},\;G_{k}=\frac{Z(\omega )}{\omega -Z\left(\omega \right)t_{k}}.$$

The Green’s function (82) is always diagonal in k-space for translationally invariant Hamiltonians.

The momentum-dependent spectral density

(83) A(k,ω)=−ImGk(ω+i0)/π

is the main characteristic of the electronic structure of strongly correlated systems. It contains information both about the quasiparticle energy dispersion (given by poles of $G_{k}\left(z\right)$) and about the incoherent bands (corresponding to the branch cuts of the Green’s function). It is proportional to ARPES intensity in the so-called direct-transitions limit (see Section 5.4).

Let us study the property of the Green’s function (82). We rewrite it in the form

(84) $$G_{k}=\frac{1}{\omega -t_{k}-\sum \left(\omega \right)},\;\sum \left(\omega \right)=U\bar{n}\left[1+\frac{U\left(1-\bar{n}\right)}{\omega -U\left(1-\bar{n}\right)}\right].$$

It is clear that the self-energy ∑(ω) is due to the interaction. Only the static (ω-independent) part of it, $\sum_{SCF}=U\bar{n}$, is taken into account in the mean-field approximation. Then, the Green’s function has a simple pole form

(85) $$G_{k,SCF}=\frac{1}{\omega -t_{k}-U\bar{n}},$$

the pole position being the one-particle energy of the mean-field Hamiltonian (68). The spectral density has a single delta-functional peak

$$A_{SCF}\left(k,\omega \right)=\delta \left(\omega -t_{k}-U\bar{n}\right)$$

with a unit weight for each spin direction.

The correlations are responsible for the dynamic part of the self-energy, which is local (k-independent) in the Hubbard-I approximation. J. Hubbard was the first to show that the correlations split the single mean-field band into two subbands, which are now called the low- and upper-Hubbard bands. Indeed, the Green’s function (84) has two poles

(86) $$G_{k}=\frac{Z_{1}}{\omega -\omega_{1}}+\frac{Z_{2}}{\omega -\omega_{2}},$$

where $\omega_{1,2}$ are the solutions of the equation ${\left[G_{k}\left(\omega \right)\right]}^{-1}=0$, which gives

$$\begin{array}{cc} \; & \omega^{2}-\omega \left(U+t_{k}\right)-U\left(1-\bar{n}\right)t_{k}=0, \end{array}$$

(87) $$\begin{array}{cc} \omega_{1,2} & =\frac{U+t_{k}}{2}\pm \sqrt{{\left(\frac{U+t_{k}}{2}\right)}^{2}-Ut_{k}\left(1-\bar{n}\right)}, \end{array}$$

(88) $$\begin{array}{cc} Z_{1} & =\frac{U\left(1-\bar{n}\right)-\omega_{1}}{\omega_{2}-\omega_{1}},\;Z_{2}=1-Z_{1}=\frac{\omega_{1}-U\left(1-\bar{n}\right)}{\omega_{2}-\omega_{1}}. \end{array}$$

This expression may be simplified in the strong correlation limit (70). Up to the terms of order $tU^{-1}$, we may write [16]

(89) $$\omega_{1}\approx t_{k}\left(1-\bar{n}\right),\;\omega_{2}\approx U+t_{k}\bar{n},\;Z_{1}\approx 1-\bar{n},\;Z_{2}\approx \bar{n}.$$

Now, it is clear that the two bands are separated by a gap of the order of *U*.

The low-energy Hamiltonian for the half-filled system ($\bar{n}=1$) is equivalent to an isotropic Heisenberg model

(90) $$\hat{H}=\frac{1}{2}\sum_{R,g}J_{g}{\hat{S}}_{R}{\hat{S}}_{R+g},$$

where $J_{g}=4t^{2}/U$. This was proven by the canonical transform, see Refs. [38,39,40,41] and references therein.

### 4.2. Anderson and Emery Models

The single impurity Anderson model (SIAM) Hamiltonian reads

(91) $$\begin{array}{cc} \; & {\hat{H}}_{SIAM}=\sum_{k\sigma }\varepsilon_{k}a_{k,\sigma }^{†}a_{k,\sigma }+{\hat{H}}_{f}+\hat{V}, \end{array}$$

(92) $$\begin{array}{cc} {\hat{H}}_{f} & \equiv \varepsilon_{f}\sum_{m}{\hat{n}}_{m}^{f}+\frac{U}{2}\sum_{m\neq m^{\prime }}{\hat{n}}_{m}^{f}{\hat{n}}_{m^{\prime }}^{f},\;\hat{V}\equiv \sum_{k,m,\sigma }V_{k,m,\sigma }f_{m}^{†}a_{k,\sigma }+h.c., \end{array}$$

where the first term of ${\hat{H}}_{SIAM}$ describes a band of uncorrelated electrons, the second term ${\hat{H}}_{f}$ is a generalized single-site Hubbard term, which is the Hamiltonian of a transition metal impurity that has a localized degenerate level with the single-partical energy $\varepsilon_{f}$ and strong Coulomb repulsion *U*. Operator $f_{m}^{†}$ creates an electron in a localized state, *m* is the set of quantum numbers that characterize the state (e.g. combination of orbital and spin projection numbers for *d*-electrons or total moment *j* projection for *f*-electrons), ${\hat{n}}_{m}^{f}\equiv f_{m}^{†}f_{m}$. The last term $\hat{V}$ represents the hybridization between the localized and delocalized states. The Hamiltonian ${\hat{H}}_{SIAM}$ (91) was first introduced in Ref. [42] for the explanation of the existence of localized magnetic moments in dilute magnetic alloys (see also Ref. [43]). The moments are localized on *d*-ion impurities in non-magnetic metals and are due to strong correlations within the *d*-shell of the ions. The model was intensively studied and allowed to explain magnetic and transport properties of the dilute magnetic alloys. In the limit of small s-d mixing, it was shown to be equivalent to the model introduced by Kondo [44]. The equivalence was proved by Schrieffer and Wolff [45] by means of canonical transform (see Section 3). In the beginning of the 1980s, exact solutions for the Anderson and Kondo models were found (see the review of Tsvelick and Wiegmann [46]). These solutions are valid only for the impurities in “good” metals, where the Fermi energy of uncorrelated electrons $E_{F}$ is the largest energy parameter of the model, and the electron spectrum may be linearized $\varepsilon \left(k\right)\approx v_{F}\left(k-k_{F}\right)$. Then, the model is reduced to a one-dimensional problem and the Bethe Ansatz approach may be applied [46].

In Ref. [47], the Anderson model was applied for the description of *d*-ion impurities in semiconductors. In the following works, it was widely used for the description of diluted magnetic semiconductors.

The interest in SIAM increased considerably when the dynamical mean-field theory (DMFT) [48,49] was formulated. The authors of Ref. [50] showed that the Hubbard model in infinite dimensions may be exactly mapped onto a single-impurity Anderson model.

An important generalization of the Anderson model is the periodic Anderson model (PAM), where transition metal ions form a periodic sublattice. It is given by the Hamiltonian

(93) $${\hat{H}}_{PAM}=\sum_{k\sigma }\varepsilon_{k}a_{k,\sigma }^{†}a_{k,\sigma }+\sum_{i}\left({\hat{H}}_{f,i}+{\hat{V}}_{i}\right),$$

where i enumerates the sites of the transition metal sublattice, operators ${\hat{H}}_{f,i},{\hat{V}}_{i}$ have the form of Equation (92) with the substitution $f_{m}^{†}\to f_{m,i}^{†}$.

The discovery of high-$T_{c}$ cuprate superconductors (HTSC) [1] immediately made the low-dimensional strongly correlated systems the focus of scientific community attention. P.W. Anderson realized the importance of correlations for the physics of cuprates [51,52]. The model for the description of hole motion in the CuO${}_{2}$ planes of HTSC was proposed by Emery [27] as a generalization of the Hubbard model

(94) $${\hat{H}}_{\mathrm{Emery}}=\sum_{i,j,\sigma }\epsilon_{i,j}a_{i,\sigma }^{†}a_{j,\sigma }+\frac{1}{2}\sum_{i,j,\sigma ,\sigma^{\prime }}U_{i,j}a_{i,\sigma }^{†}a_{i,\sigma }a_{j,\sigma^{\prime }}^{†}a_{j,\sigma^{\prime }},$$

where i labels a copper or an oxygen site, the operator $a_{i,\sigma }^{†}$ creates a hole with spin index σ in the Cu($d_{x^{2}-y^{2}}$) or O($p_{x,y}$) orbitals, which are the ones most strongly hybridized. Only site diagonal terms ($\epsilon_{p,d}$, $U_{p,d}$) and nearest neighbor hopping ($\epsilon_{i,j}=\pm t$) and interaction ($U_{i,j}=V$) terms were taken into account. The parameter regime

(95) $$U_{d}\gg U_{p},\Delta_{pd}\equiv \epsilon_{p}-\epsilon_{d}\gg V,t$$

is relevant for HTSC (here B≫A should be understood as B/A≳2.5). Later, it was found that the account of the next-nearest neighbor oxygen–oxygen hopping $t_{pp}\ll t$ is necessary for a realistic description of HTSC [53,54]. The average number of holes in the unit cell of the Emery model $\langle {\hat{n}}_{d}+2{\hat{n}}_{p}\rangle =1+x$, $\left|x\right|<1$. Positive values of *x* correspond to hole-doped HTSC, whereas negative values describe electron-doped HTSC. The model with x=0 describes parent compounds, which are antiferromagnetic insulators. In this case, the low-energy spectrum of the Emery model may be described by an effective isotropic Heisenberg Hamiltonian (see, e.g., Refs. [55,56]). If one neglects correlations on oxygen sites ($U_{p}\approx 0$), the Emery model becomes a special case of the periodic Anderson model (93).

### 4.3. Spin-Fermion and t−J Models

The downfolding of the Emery model in the regime given by Equation (95) allows obtaining low-energy models with a reduced number of degrees of freedom. The ‘minimal’ Emery model that exhibits the essential properties of layered cuprates ($1/U_{d}=U_{p}=t_{pp}=0$) reads (in hole notation)

(96) $${\hat{H}}_{H}={\hat{H}}_{0}+\hat{V},\;{\hat{H}}_{0}=\Delta \sum_{r,\gamma }{\bar{p}}_{r,\gamma }^{†}{\bar{p}}_{r,\gamma },\;\hat{V}=t\sum_{R,\alpha ,\gamma }\left({\bar{p}}_{R+a_{\alpha },\gamma }^{†}{\bar{Z}}_{R}^{0\gamma }+{\bar{Z}}_{R}^{\gamma 0}{\bar{p}}_{R+a_{\alpha },\gamma }\right),$$

where the Fermi operator ${\bar{p}}_{r,\gamma }$ annihilates a hole at site r of the oxygen sublattice with spin projection index γ, and the Hubbard projection operator ${\bar{Z}}_{R}^{0\gamma }$, Equation (26), (see also Appendix C) annihilates a hole with spin index γ on a *singly occupied* copper site. The double occupancy of copper sites is thus excluded from (96). The first term, ${\hat{H}}_{0}$, includes the on-site energies ($\Delta =\epsilon_{p}-\epsilon_{d},$$\epsilon_{d}$ is taken as zero of energy), $\hat{V}$ is the *p*-*d* hybridization, α=x,−x,y,−y characterizes the direction of a nearest-neighbor vector a, and the phase factors in $\hat{V}$ are absorbed into the definition of the operators ${\bar{p}}_{r,\sigma },{\bar{Z}}_{R}^{0\gamma }$.

In the limit t/Δ≪1, further downfolding by means of a canonical transformation of operators of the form

(97) $${\hat{A}}_{eff}=\exp\left(-\hat{S}\right)\hat{A}\exp\left(\hat{S}\right)=\hat{A}+\left[\hat{A},\hat{S}\right]+\cdots \;,\;\hat{S}=-\frac{t}{\Delta }\sum_{R,\alpha =\pm x,\pm y,\gamma }\left(p_{R+a_{\alpha },\gamma }^{†}Z_{R}^{0\gamma }-Z_{R}^{\gamma 0}p_{R+a_{\alpha },\gamma }\right).$$

leads to the model

(98) $${\hat{H}}_{s-f}\approx {\hat{H}}_{0}-4\tau \sum_{R,\gamma }Z_{R}^{\gamma \gamma }+\tau \sum_{R,\alpha 1,\alpha 2,\gamma }p_{R+a_{\alpha 1},\gamma_{1}}^{†}p_{R+a_{\alpha 2},\gamma_{2}}\left(Z_{R}^{00}\delta_{\gamma_{1}\gamma_{2}}+Z_{R}^{\gamma_{2},\gamma_{1}}\right)-\tau \sum_{R,\alpha ,\gamma_{1}}Z_{R+g_{\alpha }}^{\gamma_{1}0}Z_{R}^{0\gamma_{1}}+{\hat{J}}_{s}\;,$$

(see also Ref. [54] for the notation). Here, *p* and *Z* mean transformed operators, ${\hat{J}}_{s}$ is the AFM copper–copper superexchange interaction, and g points to neighboring copper sites. The parameters are $\tau =t^{2}/\Delta$, and the AFM exchange $J\propto t^{4}/\Delta^{3}$. The model (96) is called *the spin-fermion model* [29,57,58].

As we have mentioned in a previous subsection, in the absence of doping, the Emery model is equivalent to the nearest-neighbor AFM Heisenberg model ${\hat{J}}_{s}$. An extra hole on the oxygen site forms a Zhang–Rice singlet and triplet states with a neighboring Cu site [28,29], the triplet state being ∼8τ higher in energy than the singlet. Exclusion of the triplet states leads to the t−J model [28]

(99) $${\hat{H}}_{t-J}=-t\sum_{i,g,\sigma }{\tilde{c}}_{i,\sigma }^{†}{\tilde{c}}_{i+g,\sigma }+J\sum_{i}\left(S_{i}S_{i+1}-\frac{n_{i}n_{i+1}}{4}\right)=-t\sum_{i,g}Z_{i}^{\sigma 0}Z_{i+g}^{0\sigma }+\frac{J}{2}\sum_{i}Z_{i}^{\alpha \beta }Z_{i+1}^{\beta \alpha },$$

where ${\tilde{c}}_{i,\sigma }^{†}\equiv Z_{i}^{\sigma 0}$. The model (99) describes the hole motion in the antiferromagnetic background of cuprate superconductors and the formation of a spin polaron [2,59,60,61,62,63].

### 4.4. Classification of Strongly Correlated Systems

In the seminal work of J. Zaanen, G. A. Sawatzky, and J. W. Allen [64], the transition metal compounds were classified according to the relations between the energetic parameters:

- The Coulomb interaction within the *d*-shell of the transition metal ion $U=E\left(d_{i}^{n+1}d_{j}^{n-1}\right)-E\left(d^{n}d^{n}\right)$;
- The charge-transfer energy $\Delta =E\left(d_{i}^{n+1}\underline{L}\right)-E\left(d^{n}\right)$ between the transition metal ion and surrounding ligand;
- The hopping integral *t* between a ligand and transition metal ion.

According to this work (see Figure 3 of Ref. [64]), the transition metal strongly correlated compounds may behave as:

**(A)**  **Mott Hubbard insulators:** t≪U≪Δ, then the gap value is $E_{\mathrm{gap}}∼U$; both holes and electrons move in d bands and are heavy. The one-band Hubbard model (67) describes the main physics of these systems.
**(B)**  **Charge transfer insulators:** t≪Δ≪U, then $E_{\mathrm{gap}}∼\Delta$ (and proportional to the electronegativity of the anion); holes are light (anion valence band), and electrons are heavy (*d* bands). The Emery model (94) and the periodic Anderson model (93) with an explicit account of the anion states are used for the description of this class of compounds. The high-$T_{c}$ cuprate superconductors and other cuprates are the most studied examples. Diluted magnetic semiconductors also belong to this class of compounds.
**(AB)**  **Intermediate region** t≪Δ∼U.

### 4.5. Many-Band Generalization of the Models

For a realistic description of a specific compound, the generalizations of the above models are necessary. In most compounds, several correlated states per site and the dependence of the $V_{k,m,\sigma }$ matrix element in Equation (92) on the symmetry of *m*-th orbital should be taken into account (see e.g., [65]). An account of the geometry of bonds and the symmetry of anion’s ligand orbitals is also necessary for the quantitative description of the transition metal compounds.

**Figure 12:**
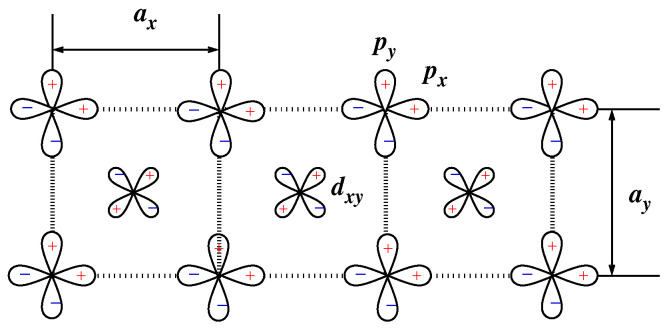
Basis of the five-band p−d model for a CuO${}_{2}$ chain.

In Ref. [66], the five-band p−d model was introduced for the electronic structure of so-called edge-shared compounds (see also [33,34,67]). The model is used for the unified consideration of magnetic properties and the optic and RIXS spectra of the compounds (e.g., [24,25,34,68,69,70,71]). The orbital basis (Figure 12) consists of a single 3$d_{xy}$ orbital on each Cu site and the 2p${}_{x}$ and 2p${}_{y}$ orbitals on each oxygen site. It is

(100) $$\begin{array}{cc} H & =\sum_{m,l,\alpha ,\sigma }t_{d,p_{\alpha }}^{ml}\left[d_{m,\sigma }^{†}p_{\alpha ,l,\sigma }+h.c.\right]+\sum_{l,l^{\prime },\alpha ,\sigma }t_{p_{\alpha },p_{\alpha }}^{ll^{\prime }}\left[p_{\alpha ,l,\sigma }^{†}p_{\alpha ,l^{\prime },\sigma }+h.c.\right]\\ \; & +U_{d}\sum_{m}n_{m,↑}^{d}n_{m,↓}^{d}+U_{p}\sum_{l,\alpha }n_{l,↑}^{p_{\alpha }}n_{l,↓}^{p_{\alpha }}+\sum_{\sigma ,\sigma^{\prime }}\left[U_{p}-\delta_{\sigma ,\sigma^{\prime }}K_{p}\right]\sum_{l}n_{l,\sigma }^{p_{x}}n_{l,\sigma^{\prime }}^{p_{y}}\\ \; & -K_{p}\sum_{l,\sigma }p_{x,l,\sigma }^{†}p_{x,l,\bar{\sigma }}p_{y,l,\bar{\sigma }}^{†}p_{y,l,\sigma }\\ \; & +K_{p}\sum_{l}\left(p_{x,l,↑}^{†}p_{y,l,↑}p_{x,l,↓}^{†}p_{y,l,↓}+p_{y,l,↑}^{†}p_{x,l,↑}p_{y,l,↓}^{†}p_{x,l,↓}\right)\\ \; & +\sum_{\sigma ,\sigma^{\prime }}\left[U_{pd}-\delta_{\sigma ,\sigma^{\prime }}K_{pd}\right]\sum_{m,l,\alpha }n_{m,\sigma }^{d}n_{l,\sigma^{\prime }}^{p_{\alpha }}-K_{pd}\sum_{l,m,\alpha ,\sigma }d_{m,\sigma }^{†}d_{m,\bar{\sigma }}p_{\alpha ,l,\bar{\sigma }}^{†}p_{\alpha ,l,\sigma }\\ \; & +K_{pd}\sum_{l,m,\alpha }\left(d_{m,↑}^{†}p_{\alpha ,l,↑}d_{m,↓}^{†}p_{\alpha ,l,↓}+p_{\alpha ,l,↑}^{†}d_{m,↑}p_{\alpha ,l,↓}^{†}d_{m,↓}\right)\\ \; & +\sum_{<m,m^{\prime }>,\sigma ,\sigma^{\prime }}U_{dd}n_{m,\sigma }^{d}n_{m^{\prime },\sigma^{\prime }}^{d}+\sum_{m,\sigma }\epsilon_{d,m}n_{m,\sigma }^{d}+\sum_{l,\alpha ,\sigma }\epsilon_{p,l,\alpha }n_{l,\sigma }^{p_{\alpha }} \end{array}$$

where *m*, $m^{\prime }$ are Cu site indices, *l*, $l^{\prime }$ are oxygen site indices, α=x,y are orbital indices for the 2$p_{x,y}$ orbitals, 〈⋯〉 is a sum over nearest neighbors, and $n^{d}$, $n^{p_{\alpha }}$ are the usual number operators for the Cu and O orbitals. Besides the one-particle on-site energies $\epsilon_{d,m}$, $\epsilon_{p,l,\alpha }$, hoppings $t_{d,p_{\alpha }}^{ml}$, $t_{p_{\alpha },p_{\alpha }}^{ll^{\prime }}$, the Hamiltonian (100) accounts for the Hubbard terms on Cu and O sites with parameters $U_{d}$ and $U_{p}$, the Hund coupling on O site ($K_{p}$), the direct Coulomb ($U_{pd}$) and exchange ($K_{pd}$) interactions between neighboring Cu and O sites, and Coulomb interaction ($U_{dd}$) between neighboring Cu sites.

When electron-lattice coupling is strong due to the Jahn–Teller effect, the orbital degrees of freedom come into play. Then, the relevant low-energy model is the so-called *Kugel–Khomskii Hamiltonian* [72]. Its main feature is the appearance of pseudo-spin values that characterize the orbital degrees of freedom. There exists a close analogy between a hole moving in the antiferromagnetic background and an electron moving in the alternating orbital environment of double exchange ferromagnets. The term “orbital polaron” was originally introduced by R. Kilian and G. Khaliullin for a quasi-particle for which the charge degree of freedom is not only coupled to orbital fluctuations, but also to the lattice [73]. Later, the term was used in studies on effective, low-energy t−J such as Hamiltonians in the field of manganites for orbital quasi-particles [74,75,76,77,78].

## 5. Response Functions Calculations and Spectroscopies

A theoretical interpretation of experimental data demands calculations of response functions. Above, we have given some examples of the calculations for finite systems, Equations (18) and (37). Below, we give more examples of the response function calculations for extended strongly correlated systems.

### 5.1. Ab Initio Ligand Field Theory to Determine Electronic Multiplet Properties

There is a fundamental problem in electronic structure theory of solids, namely the proper description of multiplet effects of local magnetic centers built up of *d* or *f* electrons, which are intrinsically many-body states, in translational invariant settings. The many-electron multiplet levels are characterized by strong Coulomb interactions, electron correlations, and spin orbit coupling. The multiplets have been well understood for many years in atomic physics. Such multiplets persist in solids, either as sharp levels in the gap of insulators or semiconductors or as resonances in metals and small gap semiconductors. The influence of the surrounding crystal on the *d* or *f* electron shell of an ion is described by a few new parameters that are traditionally called crystal field (CF) or ligand field (LF) [79,80] parameters. The knowledge of LF parameters allows describing the splittings and mixing of single-ion many-body states in a crystal and calculating the response functions, which are determined by local multiplet effects.

In the literature, one can find several approaches to calculate LF parameters. First, there are wave function quantum chemistry methods [81]. However, it is difficult for these methods to treat a periodic crystal, and they become numerically expansive for heavy ions and large systems. There exist numerous attempts in the scientific literature to calculate multiplets and LF parameters in an ab initio style and based on density functional theory (DFT) [82,83,84]. The authors of Refs. [12,85] follow a much simpler way by starting with the *non-spin-polarized* calculation using GGA functional [86]. Then, they obtain the LF parameters by a Wannier fit to the non-magnetic band structure. In Ref. [85], the LF parameters serve as input for an exact diagonalization computer program ELISA (electrons localized in single atom) to calculate the response functions sensible to local multiplet effects, i.e., electron paramagnetic resonance (EPR), optical spectroscopy, inelastic neutron scattering (INS), X-ray absorption, and X-ray magnetic circular dichroism (XAS and XMCD) as well as resonant inelastic X-ray scattering.

When RIXS experiment exploits a resonance on a core level of a transition metal ion, its intensity is given by the formula

(101) $$I\left(\omega \right)=\sum_{f}\delta \left(\omega_{in}-\omega_{out}-\left(E_{f}-E_{i}\right)\right){\left|A_{f}\right|}^{2}$$

where $\omega =\omega_{in}-\omega_{out}$ is the energy transfer, the indices *i* and *f* denote initial and final states, respectively, and with the scattering amplitude

(102) $$A_{f}=A_{f}\left(\omega_{in}\right)=\sum_{m}\frac{\langle f|{\vec{E}}_{in}\cdot \vec{r}|m\rangle \langle m|{\vec{E}}_{out}\cdot \vec{r}|0\rangle }{\omega_{in}-\left(E_{m}-E_{i}\right)+i\Gamma }$$

where we sum over all intermediate states *m*, and ${\vec{E}}_{in},{\vec{E}}_{out}$ are polarization vectors of incoming and outgoing X-ray radiation.

The optical absorption spectra are calculated by using the approach of Sugano and Tanabe [87], where the *d*-*d* transitions between two states *a* and *b* become possible by combining a parity changing perturbation $V_{\mathrm{odd}}$ with the dipole operator $\vec{P}=q\cdot \vec{r}$ to give the transition probability by

(103) $$W={\left|\frac{2}{\Delta E}\langle a|V_{\mathrm{odd}}\vec{P}|b\rangle \right|}^{2}\;,$$

where ΔE is the energy difference between the given configuration with an incomplete *d* shell and the first excited configuration with odd parity.

To calculate the optical and X-ray spectra, the dipole transition probabilities are calculated in the ELISA code as it was published for XAS and XMCD [88].

### 5.2. Spin-Hamiltonians and Magnetic Response

As we have already mentioned, in strongly correlated systems, charge and spin degrees of freedom are well separated in energy. For insulators, the spin and phonon excitations have the lowest energy and thus are responsible for thermodynamic properties. Magnetic properties are described by effective spin-Hamiltonians. Theoretical determination of parameters of the spin-Hamiltonians is the application of Löwdin downfolding to the generalized Hubbard model. The downfolding for magnetic impurities in non-magnetic materials is performed in three steps [89]: First, the virtual hoppings of electrons between the impurity ion and surrounding ligands are eliminated, and one obtains an effective ligand-field Hamiltonian. In the second step, one takes into account the fact that the largest part of the LF Hamiltonian (usually, the cubic splitting) is smaller than the remaining Coulomb interactions. Thus, an effective Hamiltonian for the lowest multiplet is obtained. Finally, the couplings of the ground state manifold with higher levels due to the smallest low-symmetry LF terms and by the spin-orbit interaction are eliminated. Thus, it is possible to obtain an analytical closed expression which connects the parameters of the microscopic Hamiltonian of the generalized Hubbard model with the parameters of the effective spin Hamiltonian [85,89,90].

Analogous downfolding is possible for the extended systems where magnetic ions form a regular sublattice. We have already mentioned the equivalence of the Heisenberg model and the low-energy behavior of the half-filled Hubbard model [38,39,40,41] and of the undoped Emery model (e.g., [55,91,92] and references therein). The account of spin-orbit interaction allows obtaining anisotropic terms [93,94,95].

Now, it becomes a standard to obtain the parameters of a spin-Hamiltonian from the spin-density-functional calculations. Comparison of total energies of different magnetic configuration allows finding the exchange values. Here, we cite only a few examples of such determination of parameters [96,97,98] just for the illustration of the method. In most cases, the density-functional theory (DFT) calculations do not provide exact values of the exchange interactions, but they allow establishing the hierarchy of the interactions. This is very important for the compounds with competing frustrated interactions. The refinement of the parameters is then possible by comparison with an experiment. Fitting of the inelastic neutron scattering (INS) spectra within the linear spin-wave theory may be ambiguous. It was the case of the quasi-one-dimensional ${\mathrm{LiCu}}_{2}O_{2}$ edge-shared cuprate compound. First, the INS spectrum of ${\mathrm{LiCu}}_{2}O_{2}$ was interpreted in terms of an antiferromagnetic $J_{1}-J_{2}$ model [99], but the DFT calculations and other experimental evidence has shown that the main interactions along the chain are *ferromagnetic* nearest neighbor $J_{1}$ and antiferromagnetic next nearest neighbor $J_{2}$ [100,101]. Both sets of parameters explain the measured INS spectrum.

Once the spin-Hamiltonian is established, all the magnetic properties of the compound may be described. For example, to model the temperature dependence of the magnetic susceptibility χ(T), the open-source program code HTE10 may be used [102,103,104]. It provides the tenth-order high-temperature expansion (HTE) of a general Heisenberg model with up to four different exchange parameters $J_{1}$, $J_{2}$, $J_{3}$, and $J_{4}$. The tenth-order HTE is indispensable for systems where the scale of the exchange interactions JS(S+1) is comparable to or exceeds the scale of thermal energy in the entire range of measurement temperatures [71,105,106]. Since the maximal measurement temperature is several hundred Kelvins under normal laboratory conditions, it has to be used in the cases where the exchange energy values (i.e., $J/k_{B}∼100$ K) are of comparable order and where the susceptibility *does not* obey the Curie–Weiss law that follows from the second-order HTE. The Curie–Weiss law fitting of experimental data, which is a common practice, may lead to false estimates of magnetic interactions in such a material [105]. The program HTE10 calculates the *exact coefficients* $c_{n}$ of the normalized susceptibility per one spin calculated in the tenth-order of HTE,

(104) $$\chi_{\mathrm{HTE}}\left(T\right)=\sum_{n=1}^{10}\frac{c_{n}}{T^{n}}$$

and Padé approximants (ratios of two polynomials of *m*-th and *n*-th order, $P_{m}\left(T\right)$ and $P_{n}\left(T\right)$, respectively) $\chi_{\mathrm{HTE}}\left(T\right)\approx \left[m,n\right]=P_{m}\left(T\right)/P_{n}\left(T\right)$, m+n≤10. The Padé approximants allow extending the region of validity of the HTE [103].

### 5.3. Electron Energy Loss Spectroscopy

What is actually measured in transmission electron energy loss spectroscopy (EELS) experiments is the partial cross section [107,108] that may be decomposed into an amplitude factor and a dynamic structure factor

$$\frac{d^{2}\sigma }{d\Omega dE}=\frac{4}{{\left(a_{0}\right)}^{2}q^{4}}S\left(q,\omega \right).$$

The dynamic structure factor characterizes the linear response of the whole electronic system on *longitudinal* electric fields with the momentum q and frequency ω (the ionic contribution may be neglected for the considered frequency range of the order of several eV). Pronounced peaks in S(q,ω) are related with charge excitations: plasmons and excitons (sometimes all of them are called “excitons” [109]). The dynamic structure factor is related to the density–density correlation function

(105) S(q,ω)≡12πN∫−∞∞dte−iωtn^q(t)n^−q(0)=1π1exp(−βω)−1ImN(q,ω),

where β is the inverse temperature,

(106) $${\hat{n}}_{q}=\frac{1}{\sqrt{N}}\sum_{r,s}\exp\left(-i\mathrm{qr}\right)\left(a_{r,s}^{†}a_{r,s}-\langle a_{r,s}^{†}a_{r,s}\rangle \right)$$

is the electronic density operator in the localized basis, the summation runs over all lattice sites r and orbital sorts *s*, and $\langle \ldots \rangle$ means the thermodynamic average. For βω≫1, we have

(107) S(q,ω)≈−1πImN(q,ω),whereN(q,ω)≡n^q|n^−q

is the retarded Green’s function that defines the inverse dielectric function

(108) $$\varepsilon^{-1}\left(q,\omega \right)=1+\frac{4\pi e^{2}}{v_{c}q^{2}}N\left(q,\omega \right),$$

with $v_{c}$ being the volume of the unit cell, and *e* is the electronic charge. The function N(q,ω) describes the response to the *unscreened* external potential. The response to the total, *screened* potential is given by the function [110]

(109) $$N_{s}\left(q,\omega \right)=\varepsilon \left(q,\omega \right)N\left(q,\omega \right),$$

In the diagrammatic language, the linear response to the total field may be expressed by the polarization operator where only irreducible graphs (which do not contain the contribution of the macroscopic electric field) should be taken into account [111,112]. Combining Equations (108) and (109), we express the dielectric permittivity via $N_{s}\left(q,\omega \right)$

(110) $$\varepsilon \left(q,\omega \right)=1-\frac{4\pi e^{2}}{v_{c}q^{2}}N_{s}\left(q,\omega \right).$$

Substituting ε(q,ω) from Equation (110) to Equation (109), we obtain the relation

(111) $$N\left(q,\omega \right)=\frac{N_{s}\left(q,\omega \right)}{1-\frac{4\pi e^{2}}{v_{c}q^{2}}N_{s}\left(q,\omega \right)}$$

which is *exact* for q→0, as it was shown in Ref. [111]. The density response function $N_{H}\left(q,\omega \right)$ calculated within a generalized Hubbard model is an approximation to $N_{s}\left(q,\omega \right)$ [113]. In other words, it describes the motion of transverse (or “mechanical” by terminology introduced in Section 2.2.2 of the Agranovich and Ginzburg book [109]) excitons. The transverse (“mechanical”) excitons are excitations that correspond to poles of dielectric permittivity, Equation (110), zeros of the inverse dielectric function, Equation (108), are determined by short-range interactions.

Using the spectral representation, we may write

(112) Ns(q,z)=∫0∞−1πImNs(q,ω′)2ω′dω′z2−ω′2]=∫0ω0+∫ω0∞=NH(q,z)+N∞(q,z).

Here, we bear in mind that the Hubbard model contributes to transitions in the low frequency region $\omega <\omega_{0}$ with $\omega_{0}$ of the order of the bandwidth, and the electrons of the rest of the solid are excited only at higher energies. In zero approximation, we may assume that in the frequency region $\omega >\omega_{0}$, the electronic polarization of the rest of the solid follows the external field immediately $N_{\infty }\left(q,z\right)\approx N_{\infty }\left(q,0\right).$ In other words, the Hubbard model is embedded into the medium with dielectric permeability $\varepsilon_{\infty }\left(q\right)=1-\frac{4\pi e^{2}}{v_{c}q^{2}}N_{\infty }\left(q,0\right).$ In fact, $\varepsilon_{\infty }$ may have its own dispersion and may be quite anisotropic for a layered or quasi-one-dimensional compound. In principle, it should be taken from, e.g., LDA calculations (we have assumed that the rest of the solid is uncorrelated) or from the experiment. It is obvious that the peak positions of the loss function

(113) L(q,ω)≡−Imε−1(q,ω)

and their intensity strongly depend on the value of $\varepsilon_{\infty }\left(q\right)$. Usually, one neglects the q-dependence and the anisotropy of $\varepsilon_{\infty }$, but it is a crude approximation, as well as another one which assumes ε(q,0)=const. For a quantitative description of EELS experiments, the detailed knowledge of $\varepsilon_{\infty }\left(q\right)$ is necessary. Then, the total dielectric function and its inverse are

(114) $$\varepsilon \left(q,\omega \right)=\varepsilon_{\infty }-\frac{4\pi e^{2}}{v_{c}q^{2}}N_{H}\left(q,\omega \right)\equiv \varepsilon_{\infty }\varepsilon_{H},\;\varepsilon^{-1}\left(q,\omega \right)=\varepsilon_{\infty }^{-1}\varepsilon_{H}^{-1}.$$

In Ref. [56], the problem of dielectric response in the strong coupling regime of a charge-transfer insulator was considered. An approach that starts from the correlated paramagnetic ground state with strong antiferromagnetic fluctuations was proposed. A set of coupled equations of motion for the two-particle Green’s function was obtained and approximately solved by means of the projection technique. The solution is expressed by a two-particle basis that includes the excitonic states with electron and hole separated at various distances. The method was applied to the multiband Hubbard (Emery) model that describes layered cuprates. It was shown that strongly dispersive branches exist in the excitonic spectrum of the ’minimal’ Emery model ($1/U_{d}=U_{p}=t_{pp}=0$). For this purpose, the downfolding to the spin-fermion model, Equation (98), was performed using the canonical transform, Equation (97), for the Hamiltonian and for the density operator, Equation (106). Then, the motion of electrons and holes in the effective Hamiltonian was considered. The exciton spectrum dependencies were analyzed on finite oxygen hopping $t_{pp}$ and on the value of on-site repulsion on oxygen, $U_{p}$.

### 5.4. Angle-Resolved Photoemission Spectroscopy

It is commonly believed that the intensity of the angle-resolved photoemission spectra (ARPES) is proportional to the one-particle spectral function [114], which is an imaginary part of the retarded Green’s function [cf. Equation (84)] divided by −π

(115) A(k,ω+i0)=−ImGk(ω+i0)/π,Gk(ω)=1/ω−εk−∑k,ω,

(116) $$\begin{array}{cc} {\langle \;\langle a_{k_{1}}|a_{k_{2}}^{†}\rangle \;\rangle }_{\omega } & \equiv -i\int_{0}^{\infty }\langle \left\{a_{k_{1}}\left(t\right),a_{k_{2}}^{†}\left(0\right)\right\}\rangle e^{i\omega t}dt=\delta_{k_{1},k_{2}}G_{k_{1}}\left(\omega \right), \end{array}$$

where $a_{k,s,\alpha }$ annihilates an electron in a bulk Bloch state, $\left\{\hat{A},\hat{B}\right\}\equiv \hat{A}\hat{B}+\hat{B}\hat{A}$, the time-dependent operator $\hat{A}\left(t\right)$ is $\hat{A}\left(t\right)=\exp\left(it\hat{H}\right)\hat{A}\exp\left(-it\hat{H}\right)$, and the angular brackets denote the ground state or thermodynamic average, Equation (72).

In Ref. [115], it was shown that this is the case only for one- and two-dimensional systems with a negligible dispersion normal to the surface. However, the actual crystals are three-dimensional, and the ARPES intensity (i.e., the steady radial photocurrent of electrons emerging from the solid along the observation direction defined by the unit vector $\hat{q}$ with energies between *E* and E+dE) is proportional to the spectral function of a more complicated Green’s function

(117) Aq^,ω=−1πImGq^,ω+i0,Gq^,ω=C^|C^†ω,

where the operator

(118) $${\hat{C}}^{†}\left(\hat{q},E\right)\equiv \int d^{3}x{\hat{\psi }}^{†}\left(x\right)\chi \left(x,\hat{q},E\right)$$

creates an electron in a state with the wave function

(119) $$\chi \left(x,\hat{q},E\right)=\left\{\begin{array}{cc} \hat{O}\left(x\right)\varphi_{>}^{*}\left(x,\hat{q},E\right),\; & x\;\mathrm{inside}\;\mathrm{the}\;\mathrm{crystal}\\ =0, & \mathrm{otherwise}. \end{array}\right.$$

Here, $\varphi_{>}$ is the low-energy electron diffraction (LEED) wave function, $\hat{O}\left(x\right)$ is the operator of electron–light coupling. The function χ(r) decays into the solid owing to the spatial decay of the LEED function, and, at the same time, it rapidly vanishes in the vacuum owing to the confinement of the initial states.

It was shown how the spectra depend on physical properties of the initial and final states of the photoemission process. Both kinds of states are solutions of the Schrödinger equation with the same Hamiltonian. For the initial states, it is necessary to find the Green’s function of the *semi-infinite crystal*. In the description of final states, the inelastic scattering due to electron–electron interaction in the propagation of the outgoing electron may be taken into account phenomenologically by introducing an absorbing optical potential into the effective Schrödinger equation for the function $\varphi_{>}\left(x,\hat{q},E\right)$ [116,117,118].

## 6. Application of the Methods to Specific Material Families

In this section, we briefly outline applications of the above methods for studies of strongly correlated materials.

### 6.1. High-$T_{c}$ Cuprate Superconductors

In cuprate materials, the most important features of the electronic structure are a large hybridization of O2p and Cu3d states in the pdσ-band and a strong local Coulomb repulsion on Cu3d states in the CuO${}_{2}$ plane. As we have already mentioned in Section 4.2, the generalized Hubbard model was proposed by Emery [27], Equation (94). The hopping parameters *t*, $t_{pp}$ were taken from DFT band-structure calculations that were regarded as equivalent to applying mean-field theory to the model. Hubbard repulsion parameters were derived from photoemission and optical experiments in Refs. [119,120,121]. Later, the calculations with the constrained-density-functional approach [122,123] confirmed the obtained values. The importance of the account of Cu-O direct exchange $K_{pd}$ was pointed out in Ref. [124].

Within the Emery model, the hole spectrum [53,54,125,126], optical conductivity [127] and EELS [56] were studied. The mechanism of superconductivity was proposed in Ref. [128].

The t−J model (see Section 4.3) obtained by downfolding of the Emery model allowed describing most of the low-energy physics of the high-$T_{c}$ superconductors. A comprehensive review is given in the book [2].

### 6.2. Edge-Shared Cuprates

The electronic structure close to Fermi energy of these compounds is defined by Cu 3d and oxygen 2p states in CuO${}_{2}$ chains (Figure 12). As we have noted in Section 4.5, the electronic structure of the chain is described by the five-band p−d model, Equation (100). The magnetic part of its spectrum is well described by the one-dimensional spin-1/2 $J_{1}$-$J_{2}$ Heisenberg model.

The ESC compounds represent a particular class of quantum magnets in which the local geometry gives rise to competing nearest ferromagnetic (FM) or antiferromagnetic (AFM) exchange coupling $J_{1}$ and frustrating antiferromagnetic next-nearest neighbor $J_{2}$ superexchange couplings.

In this rich family, one of the most studied compounds is Li${}_{2}$CuO${}_{2}$. The INS studies reported in Ref. [68] supplemented by the DFT calculations and exact diagonalization studies of Cu${}_{n}$O${}_{2n+2}$-clusters (*n* = 5, 6) allowed establishing a set of consistent parameters of the five-band p−d model that describe optical, EELS, O 1s XAS-spectral data [24,129], RIXS spectra [34,70], value of the magnetic saturation field [130], and temperature dependence of magnetic susceptibility [105].

## 7. Concluding Remarks: Building of a Microscopic Model for a Description of a Specific Material

We have outlined a realistic strategy in the description of transition metal compounds.

**Figure 13:**
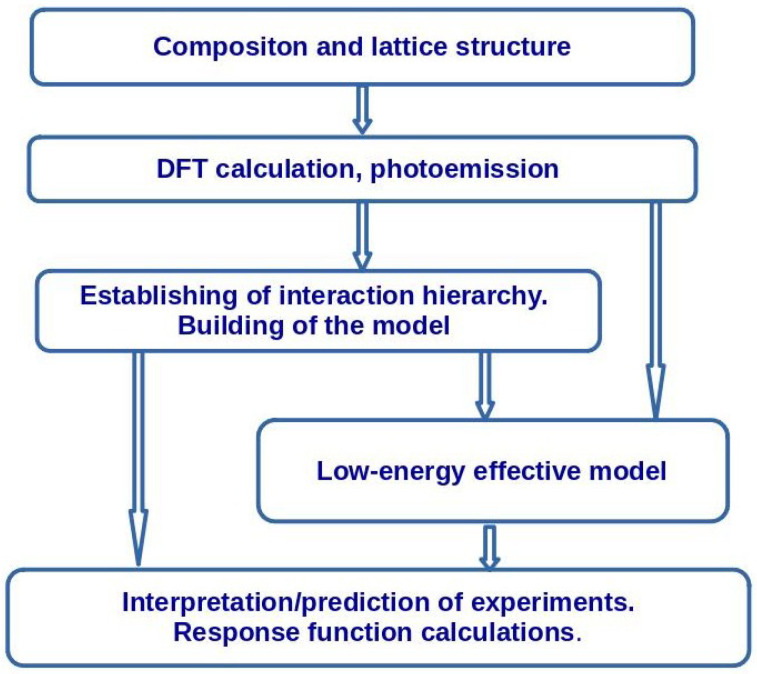
Scheme of the building of a microscopic model for a description of a specific material.

The common steps of the model building are outlined in Figure 13. First, state-of-the-art density functional theory calculations should be performed for the given composition and the crystal structure. Numerous computer codes are available for this purpose. We mention here the most popular codes: the Vienna ab initio simulation package (VASP) [131,132], WIEN2k [133], and FPLO [134]. A comparison of accuracy and an extensive list of the DFT codes may be found in Refs. [135,136]. If it is difficult to make a DFT calculation (e.g., because of a large unit cell or in the case of modeling of an impurity), Harrison’s model (see Appendix D) [137] may be used.

Photoemission spectra provide an additional input that allow estimating the value of the Hubbard repulsion *U* for the transition metal ions.

On the base of the DFT calculations and photoemission data, the hierarchy of interactions in the compound may be established. Then, the generalized many-band Hubbard model is formulated. This model allows describing the electronic structure of the system on the energy scale of several eV. The general features of charge response (approximate energies of charge-transfer transitions) may be estimated on this step.

For the calculation of magnetic response and of thermodynamic properties, one needs to pass to the low-energy scale (about onetenth or one one-hundredth of eV) using Löwdin downfolding, canonical transform or a mapping of models using energy spectra of small clusters. In previous Section 2.1 and Section 2.2, we have given examples of mapping of low-energy spectra of Hubbard models onto the Heisenberg model. These methods allow connecting the parameters of the low-energy model with the parameters of the initial Hubbard model.

We have also shown that the detailed calculations of charge response should take into account the magnetic state of the system.

## Figures and Tables

**Figure 7:**
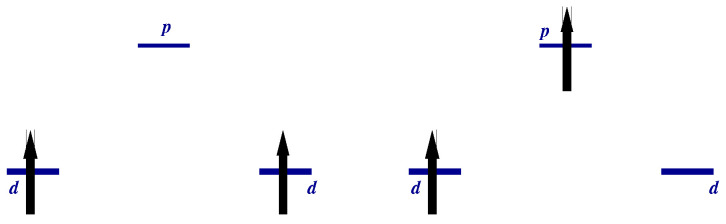
Scheme of $|t_{d}\rangle$ and $|ZRT\rangle$ states (33),(34) in a “Cu-O-Cu” molecule.

## Abbreviations

The following abbreviations are used in this manuscript:

MDPI	Multidisciplinary Digital Publishing Institute
AFM	Antiferromagnetic
ARPES	Angle-resolved photoemission spectroscopy
CF	Crystal field
DFT	Density-functional theory
DMFT	Dynamical mean-field theory
EELS	Electron energy loss spectroscopy
EPR	electron paramagnetic resonance
ESC	edge-shared cuprates
GGA	Generalized gradient approximation
HTE	High-temperature expansion
HTSC	high-$T_{c}$ cuprate superconductors
INS	Inelastic neutron scattering
LDA	Local density approximation
LEED	Low-energy electron diffraction
LF	Ligand field
PAM	Periodic Anderson model
RIXS	Resonance inelastic X-ray scattering
SIAM	Single impurity Anderson model
XAS	X-ray absorption
XMCD	X-ray magnetic circular dichroism

## Appendix A. Details of “Cu-O-Cu” Cluster Spectrum

### Appendix A.1. Spectrum in the Singlet Sector

The Hamiltonian matrix in the subspace of even singlets (27)–(29) is

(A1) $${\hat{H}}_{s}=\left(\begin{array}{ccc} 0 & t\sqrt{2} & 0\\ t\sqrt{2} & \Delta & 2t\\ 0 & 2t & 2\Delta \end{array}\right),$$

its eigenvalues are

(A2) $$\begin{array}{cc} E_{0} & =\Delta -2\sqrt{Q}\left(\frac{\sqrt{3}}{2}\cos\frac{\varphi }{3}+\frac{1}{2}\sin\frac{\varphi }{3}\right),\;E_{1}=\Delta +2\sqrt{Q}\sin\frac{\varphi }{3}, \end{array}$$

(A3) $$\begin{array}{cc} E_{2} & =\Delta +2\sqrt{Q}\left(\frac{\sqrt{3}}{2}\cos\frac{\varphi }{3}-\frac{1}{2}\sin\frac{\varphi }{3}\right), \end{array}$$

(A4) $$\begin{array}{cc} \phi & =\arcsin\frac{R}{\sqrt{Q^{3}}},\;Q=\frac{1}{3}\left(\Delta^{2}+6t^{2}\right),\;R=-t^{2}\Delta . \end{array}$$

It is instructive to expand the above expressions in powers of t/Δ to reproduce the perturbative expansion up to the fourth order

(A5) $$E_{0}\approx -\frac{2t^{2}}{\Delta },\;E_{1}\approx \Delta -\frac{2t^{2}}{\Delta }\left(1-\frac{6t^{2}}{\Delta^{2}}\right),\;E_{2}\approx 2\Delta +\frac{4t^{2}}{\Delta }-\frac{12t^{4}}{\Delta^{3}}.$$

The matrix (A1) has tridiagonal form, and its eigenvectors are

(A6) $$|f\rangle =\sum_{j=0}^{2}\alpha_{j}^{f}|j\rangle ,\;\alpha_{j}^{f}=\frac{1}{A_{f}}P_{j}\left(E_{f}\right),$$

where $A_{f}^{2}=\sum_{j=0}^{2}{\left|P_{j}\left(E_{f}\right)\right|}^{2},$ the basis states $|j=0,1,2\rangle$ are given by Equations (27)–(29) correspondingly, and $P_{j}\left(E\right)$ are polinoms, given by the recursion

(A7) $$EP_{n}\left(E\right)=a_{n}P_{n}\left(E\right)+b_{n}P_{n-1}\left(E\right)+b_{n+1}P_{n+1}\left(E\right),$$

with the initial conditions $P_{0}=1,$$P_{-1}=0$; $a_{n}=H_{s,n,n}$ are diagonal, and $b_{n}=H_{s,n,n+1}$ are off-diagonal elements of the matrix (A1), thus

(A8) $$P_{1}\left(E\right)=\frac{1}{b_{1}}\left(E-a_{0}\right),\;P_{2}\left(E\right)=\frac{1}{b_{2}}\left[\left(E-a_{1}\right)\frac{E-a_{0}}{b_{1}}-b_{1}\right],$$

Up to the second order, we have for the eigenvector matrix

$$∥\alpha_{j}^{f}∥=\left(\begin{array}{ccc} 1-y^{2} & -\sqrt{2}y & \sqrt{2}y^{2}\\ -\sqrt{2}y & -1+3y^{2} & 2y\\ \sqrt{2}y^{2} & 2y & 1-2y^{2} \end{array}\right),$$

here y≡t/Δ.

The odd singlet (31) has the energy $E_{ZRSa}=\Delta$

### Appendix A.2. Triplet Sector

The energy of even triplet is $E_{ts}=\Delta .$ The Hamiltonian matrix in the subspace of odd triplets (33) and (34) is

(A9) $${\hat{H}}_{t}=\left(\begin{array}{cc} 0 & t\sqrt{2}\\ t\sqrt{2} & \Delta \end{array}\right),$$

its eigenvalues are

(A10) $$\begin{array}{cc} E_{t,0} & =\frac{\Delta }{2}\left(1-R_{t}\right)\approx -\frac{2t^{2}}{\Delta }+\frac{4t^{4}}{\Delta^{3}}, \end{array}$$

(A11) $$\begin{array}{cc} E_{t,1} & =\frac{\Delta }{2}\left(1+R_{t}\right)\approx \Delta +\frac{2t^{2}}{\Delta }-\frac{4t^{4}}{\Delta^{3}}, \end{array}$$

where $R_{t}\equiv \sqrt{1+8{\left(t/\Delta \right)}^{2}}$. The eigenvectors are

(A12) $$\begin{array}{cc} |Gt\rangle & =\cos\phi_{t}|t_{d}\rangle +\sin\phi_{t}|ZRT\rangle , \end{array}$$

(A13) $$\begin{array}{cc} |T1\rangle & =\sin\phi_{t}|t_{d}\rangle -\cos\phi_{t}|ZRT\rangle , \end{array}$$

where

(A14) $$\cos\phi_{t}=\frac{1}{\sqrt{2}}\sqrt{1+\frac{1}{R_{t}}}\approx 1-{\left(\frac{t}{\Delta }\right)}^{2},\;\sin\phi_{t}=\frac{1}{\sqrt{2}}\sqrt{1-\frac{1}{R_{t}}}\approx \sqrt{2}\left(\frac{t}{\Delta }\right).$$

## Appendix B. Simplification of the Result of Fourth-Order Canonical Transform

Let us simplify the expression (63)

$$\begin{array}{cc} G_{njklm} & =\frac{1}{E_{nj}E_{ml}E_{mk}}+\frac{1}{E_{nj}E_{ml}E_{nk}}+\frac{1}{E_{nj}E_{mk}E_{kl}}\\ \; & +\frac{1}{E_{nj}E_{nk}E_{kl}}-\frac{1}{E_{jk}E_{mk}E_{kl}}-\frac{1}{E_{nk}E_{jk}E_{kl}}-\frac{1}{E_{mk}E_{jk}E_{ml}}-\frac{1}{E_{nk}E_{jm}E_{ml}}\\ \; & +\frac{1}{E_{nj}E_{jk}E_{kl}}+\frac{3}{E_{nj}E_{jk}E_{ml}}-\frac{3}{E_{nj}E_{kl}E_{ml}}+\frac{1}{E_{jk}E_{kl}E_{ml}},\\ \; & =\frac{1}{E_{nj}E_{ml}}\left(\frac{1}{E_{mk}}+\frac{1}{E_{nk}}\right)+\frac{1}{E_{mk}}\left(\frac{1}{E_{nj}E_{kl}}-\frac{1}{E_{jk}E_{kl}}-\frac{1}{E_{ml}E_{jk}}\right)\\ \; & +\frac{1}{E_{nk}}\left(\frac{1}{E_{nj}E_{kl}}-\frac{1}{E_{jk}E_{kl}}-\frac{1}{E_{ml}E_{jk}}\right)+\frac{E_{ml}+3E_{kl}-3E_{jk}+E_{nj}}{E_{nj}E_{ml}E_{jk}E_{kl}}. \end{array}$$

We recall that $E_{mj}\equiv E_{m}-E_{j}$, then

(A15) $$\begin{array}{cc} G_{njklm}\; & =\frac{1}{E_{nj}E_{ml}}\left(\frac{1}{E_{mk}}+\frac{1}{E_{nk}}\right)+\left(\frac{1}{E_{mk}}+\frac{1}{E_{nk}}\right)\frac{E_{jk}E_{ml}-E_{nj}E_{ml}-E_{nj}E_{kl}}{E_{nj}E_{ml}E_{jk}E_{kl}}\\ \; & +\frac{\left(E_{m}+3E_{k}-2E_{l}-2E_{j}\right)+\left(E_{m}+3E_{k}-2E_{l}-2E_{j}\right)}{E_{nj}E_{ml}E_{jk}E_{kl}}. \end{array}$$

Now, the last term we rewrite as following

(A16) $$\frac{1}{E_{nk}}\frac{\left(E_{m}+3E_{k}-2E_{l}-2E_{j}\right)\left(E_{n}-E_{k}\right)}{E_{nj}E_{ml}E_{jk}E_{kl}}+\frac{1}{E_{mk}}\frac{\left(E_{n}+3E_{k}-2E_{l}-2E_{j}\right)\left(E_{m}-E_{k}\right)}{E_{nj}E_{ml}E_{jk}E_{kl}}.$$

Combining terms with similar denominators, we have

(A17) $$\begin{array}{cc} \; & G_{njklm}=\frac{1}{E_{nj}E_{ml}}\left(\frac{1}{E_{mk}}+\frac{1}{E_{nk}}\right)\\ \; & +\frac{1}{E_{mk}E_{nj}E_{ml}E_{jk}E_{kl}}\left\{-3E_{j}E_{l}+2E_{k}E_{m}+3E_{k}E_{l}-2E_{n}E_{k}+3E_{j}E_{k}-3E_{k}^{2}+2E_{n}E_{l}-2E_{l}E_{m}\right\}\\ \; & +\frac{1}{E_{nk}E_{nj}E_{ml}E_{jk}E_{kl}}\left\{-3E_{j}E_{l}-2E_{k}E_{m}+3E_{k}E_{l}+2E_{n}E_{k}+3E_{j}E_{k}-3E_{k}^{2}+2E_{j}E_{m}-2E_{j}E_{n}\right\}\\ \; & =\frac{1}{E_{nj}E_{ml}}\left(\frac{1}{E_{mk}}+\frac{1}{E_{nk}}\right)+\frac{1}{E_{mk}}\frac{-3E_{kl}E_{jk}+2E_{mn}E_{kl}}{E_{nj}E_{ml}E_{jk}E_{kl}}+\frac{1}{E_{nk}}\frac{3E_{kl}E_{jk}+2E_{mn}E_{jk}}{E_{nj}E_{ml}E_{jk}E_{kl}}. \end{array}$$

## Appendix C. Hubbard Projection Operators

The Hubbard projection operators are defined as

(A18) $$X_{i}^{\alpha \beta }\equiv |\alpha ,i\rangle \langle \beta ,i|,\;\alpha ,\beta =0,↑,↓,2.$$

They are related with bare fermionic and spin operators through

(A19) $$\begin{array}{cc} X_{i}^{0\sigma } & ={\tilde{c}}_{i,\sigma }^{†}=c_{i,\sigma }\left(1-n_{i,-\sigma }\right),\;X_{i}^{\alpha 2}=-\sigma c_{i,-\sigma }n_{i,\sigma }, \end{array}$$

(A20) $$\begin{array}{cc} X_{i}^{+-} & =S_{i}^{+}=c_{i,↑}^{†}c_{i,↓},\;X_{i}^{\sigma \sigma }=\frac{1}{2}+\frac{\sigma }{2}\left(c_{i,↑}^{†}c_{i,↑}-c_{i,↓}^{†}c_{i,↓}\right)=\frac{1}{2}+\sigma S_{i}^{z} \end{array}$$

Other relations are easy to obtain with the use of the main property of Hubbard operator algebra

(A21) $$X_{i}^{\alpha \beta }X_{i}^{\gamma \lambda }=\delta_{\beta \gamma }X_{i}^{\alpha \lambda }$$

that follows immediately from the definition (A18).

## Appendix D. Harrison’s Understanding of Electronic Structure

In his seminal book [137], W.A. Harrison succeeded in describing the electronic structure of a huge number of compounds, assuming the existence of an orthonormal basis of one-particle spherically symmetric functions localized on lattice sites. It is also a “minimal” basis set, i.e., one radial function suffices for the description of one electronic shell.

According to the Harrison model [137], the hopping $t_{d_{\alpha },p_{\beta }}^{ij}$ between a $d_{\alpha }$-function of metal ion ($\alpha =xy,yz,zx,x^{2}-y^{2},z^{2}$) and $p_{\beta }$-function of ligand (β=x,y,z) is expressed via direction cosines l,m,n of the direction of the vector $R_{i}-R_{j}$, and two Slater–Koster [138] parameters $V_{pd\sigma }\left(R\right),V_{pd\pi }\left(R\right)$, which depend on sorts of metal ions and on the distance $R=\left|R_{i}-R_{j}\right|$

(A22) $$V_{pdm}\left(R\right)=\eta_{pdm}\frac{\hbar^{2}r_{d}^{3/2}}{mR^{7/2}},\;\eta_{pd\sigma }=-2.95,\;\eta_{pd\pi }=1.36,$$

where the value $r_{d}$ (Å) is specific for a transition metal ion. The distance *R* is measured in Å and $t_{pdm}$ in eV and the ratio $\hbar^{2}/m=7.62$eV·Å${}^{2}$.

Often, the Harrison model is applied to strongly correlated solids before/instead of elaborated DFT calculations.
